# Immunoproteomic insights into inflammatory diseases of the critically endangered black rhinoceros (*Diceros bicornis*)

**DOI:** 10.1038/s41598-026-43055-0

**Published:** 2026-03-14

**Authors:** Molly L. Corder, Tamara Abulez, Timothy Cleland, Emanuel F. Petricoin, Weidong Zhou, Jennifer Nagashima, Michele Miller, Peter Buss, Leana Rossouw, Scott Citino, John A. Griffioen, Holly Haefele, Janine L. Brown, Steve Paris, Rebecca Dikow, Parker Pennington, Thomas P. Conrads, Nicholas W. Bateman, Joshua Davis, A. Alonso Aguirre, Budhan Pukazhenthi

**Affiliations:** 1 Center for Species Survival, Smithsonian’s National Zoo & Conservation Biology Institute, Front Royal, VA USA; 2 Environmental Science & Policy Department, George Mason University, Fairfax, VA USA; 3 Gynecologic Cancer Center of Excellence, Department of Gynecologic Surgery and Obstetrics, Uniformed Services University of the Health Sciences, Walter Reed National Military Medical Center, Bethesda, MD USA; 4 The Henry M. Jackson Foundation for the Advancement of Military Medicine Inc, Bethesda, MD USA; 5 Museum Conservation Institute, Smithsonian Institution, Suitland, MD USA; 6 Center for Applied Proteomics & Molecular Medicine, George Mason University, Manassas, VA USA; 7 South African Medical Research Council Centre for Tuberculosis Research, Division of Molecular Biology and Human Genetics, Faculty of Medicine and Health Sciences, Stellenbosch University, Cape Town, South Africa; 8 Veterinary Wildlife Services, South African National Parks, Kruger National Park, Skukuza, South Africa; 9 White Oak Conservation, Yulee, FL USA; 10 Fort Worth Zoo, Fort Worth, TX USA; 11 Fossil Rim Wildlife Center, Glen Rose, TX USA; 12 Data Science Lab, Office of the Chief Information Officer, Smithsonian Institution, Washington, DC USA; 13 Murtha Cancer Center/Research Program, Department of Surgery, Uniformed Services University of the Pathology and Laboratory Medicine, Inova Health Sciences, Bethesda, System, MD, VA USA; 14 Women’s Health Integrated Research Center, Inova Women’s Service Line, Inova Health System, Falls Church, VA USA; 15 Smithsonian-Mason School of Conservation, George Mason University, Front Royal, VA USA; 16 Present Address: Yale University, New Haven, CT USA; 17 Present Address: Colossal Biosciences, Dallas, TX USA; 18 Present Address: Department of Fish, Wildlife and Conservation Biology, Warner College of Natural Resources, Colorado State University, Fort Collins, CO USA

**Keywords:** Black rhinoceros, Conservation medicine, Proteomics, Machine learning, Ecology, Ecology, Evolution, Zoology

## Abstract

Black rhinoceros are critically endangered due to poaching in the wild (in situ). Globally, fewer than 200 animals are maintained as an ex situ insurance population. Unfortunately, the ex situ population faces major sustainability challenges from disease syndromes characterized by high inflammatory burdens and diverse manifestations of immunometabolic dysfunction, not known to be present among their wild counterparts. Overlapping ex situ disease phenotypes limit diagnostic specificity and highlight the need to define underlying disease mechanisms. In the present study, using a cohort of presumed clinically healthy and inflammatory black rhinoceros, we generated the first immunoproteomic profile of any endangered mammal species and identified 1,311 immune cell proteins. However, no significant differences were detected among clinical phenotypes. Therefore, we applied unsupervised machine learning approaches to detect molecular features suggestive of healthy versus inflammatory phenotypes. Forty-three proteins associated with inflammatory pathways were differentially expressed in a cohort of samples derived from both presumed healthy and inflammatory phenotypes. Results suggest subclinical disease may be relatively widespread ex situ, and that animals experience temporal fluctuations in inflammatory state over time. Findings implicate neutrophil degranulation and dysregulation of the oral-gut-liver axis as drivers of disease syndromes of ex situ black rhinoceros. The forty-three proteins associated with inflammatory pathways represent candidate inflammatory biomarkers to be assessed for clinical applications in future validation studies. Upon validation, these candidate biomarkers may guide management practices to strengthen long-term population sustainability.

**Supplementary Information:**

The online version contains supplementary material available at 10.1038/s41598-026-43055-0.

## Introduction

Species threatened with extinction frequently exist in small, fragmented populations in the wild which necessitates managing some animals under human care (zoological facilities; ex situ) to optimize propagation, serve as a genetic reservoir against impending extinction threats, and ultimately ensure species survival. Black rhinoceros (*Diceros bicornis*; “black rhino”) are critically endangered primarily due to poaching in the wild (in situ). The International Union for the Conservation of Nature recognizes three extant and one extinct subspecies of black rhinoceros^1^; and recent phylogeographic genomic assessments suggest extant black rhinoceros could be genetically structured into six major historic populations (Central Africa, East Africa, Northwestern Africa, Northeastern Africa, Ruvuma, and Southern Africa)^2^. Fewer than 200 individuals exist in ex situ environments globally^3,4^. The ex situ population serves as an insurance for preserving biodiversity; and the recent extinction of the western black rhinoceros subspecies (*D. b. longiceps*) in 2011 underscores the importance of maintaining viable ex situ stock to support future reintroductions^5^. Unfortunately, ex situ black rhinoceros also face substantial population sustainability challenges due to disease syndromes not known to be present among their wild counterparts. These syndromes remain largely understudied in both in situ and ex situ populations. Multiple ex situ disease phenotypes often occur concurrently^6–9^, making accurate and specific diagnosis virtually impossible. Commonly reported clinical phenotypes include periodontitis, gastrointestinal dysbiosis, systemic inflammation, and suspected liver disease (Supplemental Information S1-2)^6,8–10^. When rhinos exhibit clinical signs of disease, veterinarians intervene as appropriate with treatments (e.g., antibiotics, anti-inflammatory drugs) to improve animal health and wellbeing. Diagnostic testing to determine potential etiologies typically includes a physical examination and assessment of standard blood parameters (e.g., complete blood count, serum biochemistry) to evaluate the functional status of various organ systems and general systemic health. Less commonly but with increasing frequency, clinicians may also evaluate biomarkers for inflammation including serum amyloid A (SAA)^11–14^, C-reactive protein, and haptoglobin^11,12,15,16^. However, these tests have not fully revealed specific physiological mechanisms driving these complex disease syndromes, which likely involve multiple organ systems and physiological mechanisms.

A wide range of clinical signs associated with immune and metabolic dysfunction^6,9–11,17,18^ are commonly observed among ex situ black rhinos and may indicate complex underlying health issues. Iron overload disorder (IOD), characterized by excessive iron accumulation in the liver and other tissues, is among the most commonly reported diseases present in ex situ but not in situ black rhinos, and can only be definitively diagnosed post-mortem^8,19,20^. In humans, iron dysregulation has been linked with immune and metabolic dysfunction^21^, but relationships between dysregulated iron metabolism, liver function, inflammation, and immune function remain poorly understood in black rhinos. Previous research evaluating inflammatory^10,14,17,18^ and metabolic^9,11^ disease in ex situ managed black rhinos provided critical baseline data on the altered physiological processes. In vitro research evaluated lymphocyte responses to non-specific (mitogenic) and specific (antigenic) stimuli, known to preferentially stimulate T or B cell differentiation into functional effector cells; and showed black rhinos have the least vigorous immune cell response when compared to other rhinoceros species in human care^18^. Another study explored the potential role of corticosteroids in immune function through corticosteroid-induced suppression of lymphocyte proliferation in vitro and detected no differences across ex situ rhinoceros species^17^. Yet, a large proportion of black rhinos in human care experience pro-inflammatory states, decreased insulin sensitivity, and increased insulin levels compared to their wild counterparts^11,14^. Despite these advances, there is little information on cellular mechanisms (protein expression) of inflammatory and metabolic diseases in black rhinos.

Analyzing immune cells can offer a deeper understanding of the molecular basis of disease syndromes. Peripheral blood mononuclear cells (PBMCs) function as physiological sensors that circulate in a quiescent state, monitoring the body for immune-relevant events (i.e., injury, pathogens, and autoimmune diseases)^22–24^. Upon detection of immune threats, PBMCs undergo activation and stimulate the production of cell-specific proteins that enable the immune response^22^ and modulate target tissue gene expression. These responses include leukocyte migration, proliferation of T-cells, activation of other immune cells, stimulation of interferon pathways, release of immune-specific secretory proteins, and controlled cell death^22–24^. Therefore, characterization of the PBMC proteome could facilitate the identification of protein signatures that can improve our understanding of disease pathophysiology and lead to the discovery of candidate biomarkers that, once validated, may prove useful for disease diagnostics^22,25–27^. To this end, untargeted PBMC proteome profiling has been used to better understand autoimmune diseases^28,29^, metabolic syndrome^30^, sepsis^31^, and inflammatory disease^32^ among others^22^ in human patients.

Few studies have examined PBMC proteomes in wildlife species. PBMC proteomes have been described in Egyptian fruit bats (*Rousettus aegyptiacus*) and black flying fox bats (*Pteropus alecto*) in the context of characterizing immunological features of these infectious disease reservoirs^33,34^; and in the critically endangered European eel (*Anguilla aguilla*) in the context of environmental toxicology^35^. However, there are no reports on the proteome of PBMCs of any rhinoceros species. In the present study, we report the first-ever characterization of the PBMC proteome (“immunoproteome”)^33^ of any endangered mammal species. Data were leveraged to test the hypothesis that PBMC proteome profiles differ between rhinos with and without inflammatory disease. In the present study, two strategies were applied. First, a metadata (presumed healthy vs. inflammatory phenotype)-driven approach was attempted to (1) annotate the immunoproteome with veterinary clinical records and (2) identify differences in molecular signatures between phenotypes. We unexpectedly observed no significant differences among clinical phenotypes, prompting further investigation using a data-driven machine learning approach to identify biochemical features of individual samples using an unsupervised class discovery machine learning algorithm^36^.

This data-driven approach led to the identification of molecular pathways involved in periodontitis, gastrointestinal dysbiosis, and systemic inflammation in various tested samples, which was driven by the expression of forty-three proteins that we present as candidate inflammatory biomarkers for this species. Moreover, longitudinal analyses indicated that molecular features of individual samples switch between putative “healthy” and “inflamed” classes over time, suggesting that individual animals experience temporal fluctuations in inflammatory phenotypes over time. This study represents the most comprehensive analysis of rhinoceros immune function to date and demonstrates the value of machine learning for discovery of disease mechanisms in small populations with potential applications in understanding of diseases processes in rare species and rare diseases.

## Results

### Immunoproteome profiling

Whole blood (EDTA) samples were collected longitudinally from 27 ex situ black rhinos representing 13 southern black rhinoceros (*D. b. minor*) (6 male and 7 female) and 14 eastern black rhinoceros (*D. b. michaeli*) (7 male and 7 female) with diverse clinical histories, as determined via a health survey (Supplemental Information S1 & S2). This study population represents ~ 65% of southern and ~ 26% of eastern black rhinos managed in the United States. In total, 80 PBMC pellets were collected for untargeted proteomic profiling. Sampling occurred across 12 sampling periods with up to 4 longitudinal sampling events per individual (Supplemental Information S3). After filtering proteins with < 1% false discovery rate (FDR), removing proteins with ≥ 25% missing values, and aggregating to the median of duplicate spectral intensities, we detected 1,311 proteins in total. Protein intensities – mass spectrometry-derived measurements of ion signal strength that reflect the relative abundance of proteins within samples – were used for all bioinformatic analyses. A linear mixed effects model was unable to detect the presence of any inherent hardwired biases in the proteomic dataset based on protein intensities and metadata covariates: subspecies, sex, health status, age class, or season as fixed effects and individual, institution, and sampling period as random effects (Supplemental Information S4). Further, graphical diagnostics generated from the model residuals indicated that the assumptions of normality and homoscedasticity were satisfactorily met (Supplemental Information S4). Principal component analysis (PCA) of protein intensity data plotted by sample and colored by relevant metadata covariates did not exhibit clear clustering patterns by metadata covariates suggesting high heterogeneity within the study population (Supplemental Information S5A-G). Further, longitudinally collected samples from the same individuals did not cluster together within a PCA ordination (Supplemental Information S5A) suggesting biochemical shifts within individuals over time.

### Differential protein expression as a function of covariates

We evaluated differences in PBMC proteomic profiles based on metadata covariates: subspecies (eastern vs. southern), sex (male vs. female), and health status (inflamed vs. putative healthy phenotype). We detected no differences in the PBMC proteome expression based on metadata covariates (Supplemental Information S6; adjusted P value < 0.05 and fold change < log2(0.5) (down-regulated) and > log2(1.5) (up-regulated)) except for two proteins, H1-5 and H2AC8 in the health status comparison (inflamed vs. putative healthy phenotype), that were down-regulated in the inflammatory group.

### Serum amyloid A (SAA) and health status

After determining that PBMC proteomes were not different based on metadata covariates, we attempted to validate clinical health status designations (presumed healthy vs. inflammatory phenotype) by measuring circulating SAA values among animals with and without clinical histories of inflammatory phenotypes since SAA has been previously characterized in association with the inflammatory process of African rhinoceros species^11–14,37^. Surprisingly, no significant differences were detected in SAA values based on clinical designations via Wilcoxon rank-sum test with continuity correction (W = 50.5, P value = 0.1213). Highest SAA values detected were in presumed healthy animals, and multiple individuals in both cohorts fell within the previously published subclinical disease SAA reference range (clinically healthy (< 1 mg/L), subclinical (1–7 mg/L), and clinically abnormal (> 7 mg/L))^13^ indicating the likely presence of subclinical disease in both the presumed healthy and inflammatory groups within the ex situ population (Supplemental Information S7 & Supplementary Methods).

### Unsupervised class discovery consensus cluster analysis

**Fig. 1:**
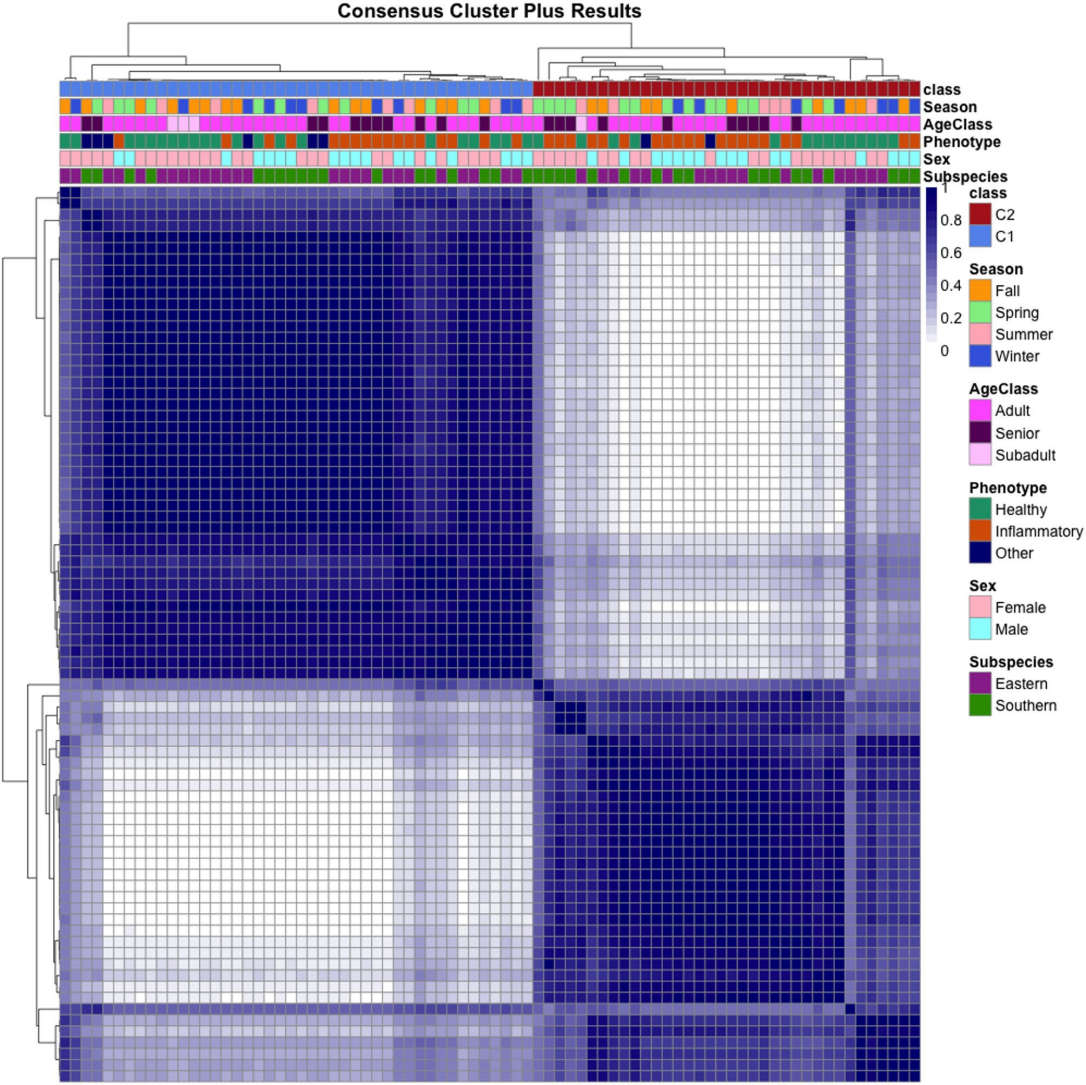
Class discovery using unsupervised machine learning. After we were unable to detect differences in PBMC profiles based on covariates (subspecies, sex, and health phenotype), we elected to apply a data-driven approach using only biochemical signatures of individual samples and no metadata information. Using an unsupervised class discovery machine learning algorithm with R package Consensus Cluster Plus, we detected two groups (k=2) within the PBMC proteome dataset. Consensus Cluster Plus (CCP) class 1 consisted of 44 samples and class 2 of 36 samples. Group membership based on individual samples was not associated with covariates (subspecies, sex, health phenotype, or age class; Supplemental Information S7E). In this squared, mirrored sample x sample matrix the same clustering dendrogram is applied to both rows and columns and is done so sample by sample (n=80 samples).

To evaluate potentially unique groups that exist within the dataset, we used data-driven unsupervised machine learning algorithms including K-means, hierarchical clustering, and consensus clustering (Supplemental Information S8A-C). Of these, consensus clustering proved to be the most robust and objective approach for class discovery based on iterative hierarchical clustering that did not require *a priori* cluster number selection, harnessed the top 25% most dynamic (variable) proteins as training data; and reproducibly identified two stable clusters that both (1) reduced noise from invariant proteins and (2) captured biologically meaningful variation within the dataset (Fig. 1). Of the 80 samples evaluated, 44 and 36 samples fell into distinct clusters based on proteomic profiles. Clusters were named Consensus Cluster Plus “CCP” class 1 (44 samples) and CCP class 2 (36 samples). Relationships between the CCP classes and covariates of interest (subspecies, sex, and health phenotype reported by animal managers) as well age class (subadult, adult, and senior) were evaluated using Fisher’s exact tests, but no significant associations were detected (Supplemental Information S8E).

### Differentially expressed proteins as a function of CCP class

**Fig. 2:**
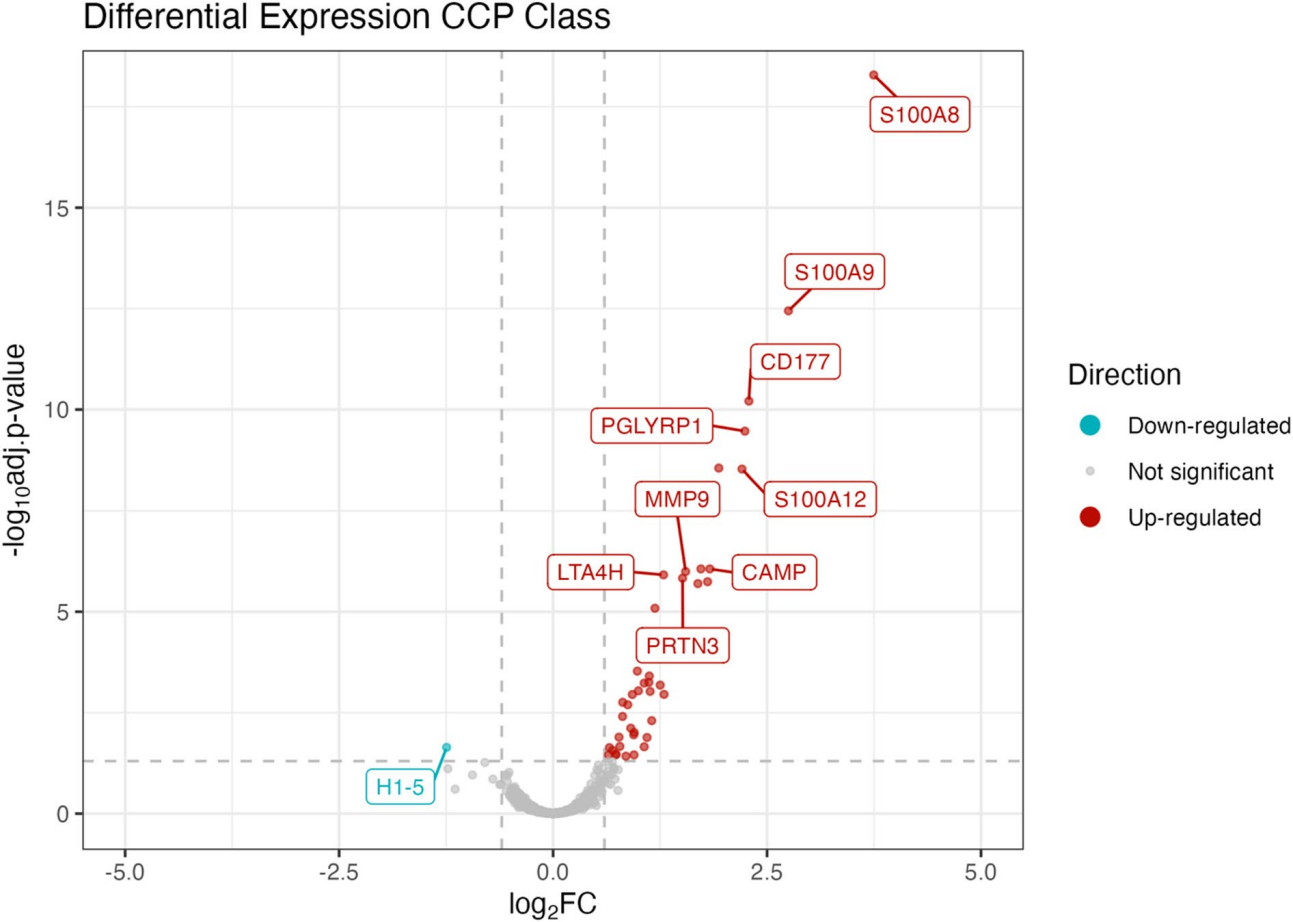
Candidate inflammatory biomarkers identified via differential expression of proteins driving CCP class differences. Log2 fold change based on CCP class 2 (putative diseased sample group) compared to CCP class 1. X-axis represents fold change: down-regulated > log2(0.5) & up-regulated < log2(1.5). Y-axis represents significance assigned to proteins with a Benjamini-Hochberg adjusted P value < 0.05.

Differential expression analysis was run between CCP classes 1 and 2 (Fig. 2) to identify proteins driving differences among naturally emerging groups (CCP classes). The CCP class differences were driven by forty-three proteins that were significantly differentially expressed (adjusted P value < 0.05) with forty-two proteins up-regulated and one protein down-regulated (fold change: down-regulated > log2(0.5) & up-regulated < log2(1.5)) in CCP class 2 (Supplemental Information S9). These forty-three proteins driving CCP class differences were later deemed candidate inflammatory biomarkers (see below). Further, we determined that PBMCs collected from the same donor often switched class designation over time, indicating animals experience temporal fluctuations in inflammatory state over time. We observed class switching in 22 out of 27 animals included in this study (Supplemental Information S10 A-C). Intra-animal variability in PBMC profiles mirrored findings reported for CCP class differential expression (data not shown).

### Functional profiling

**Fig. 3:**
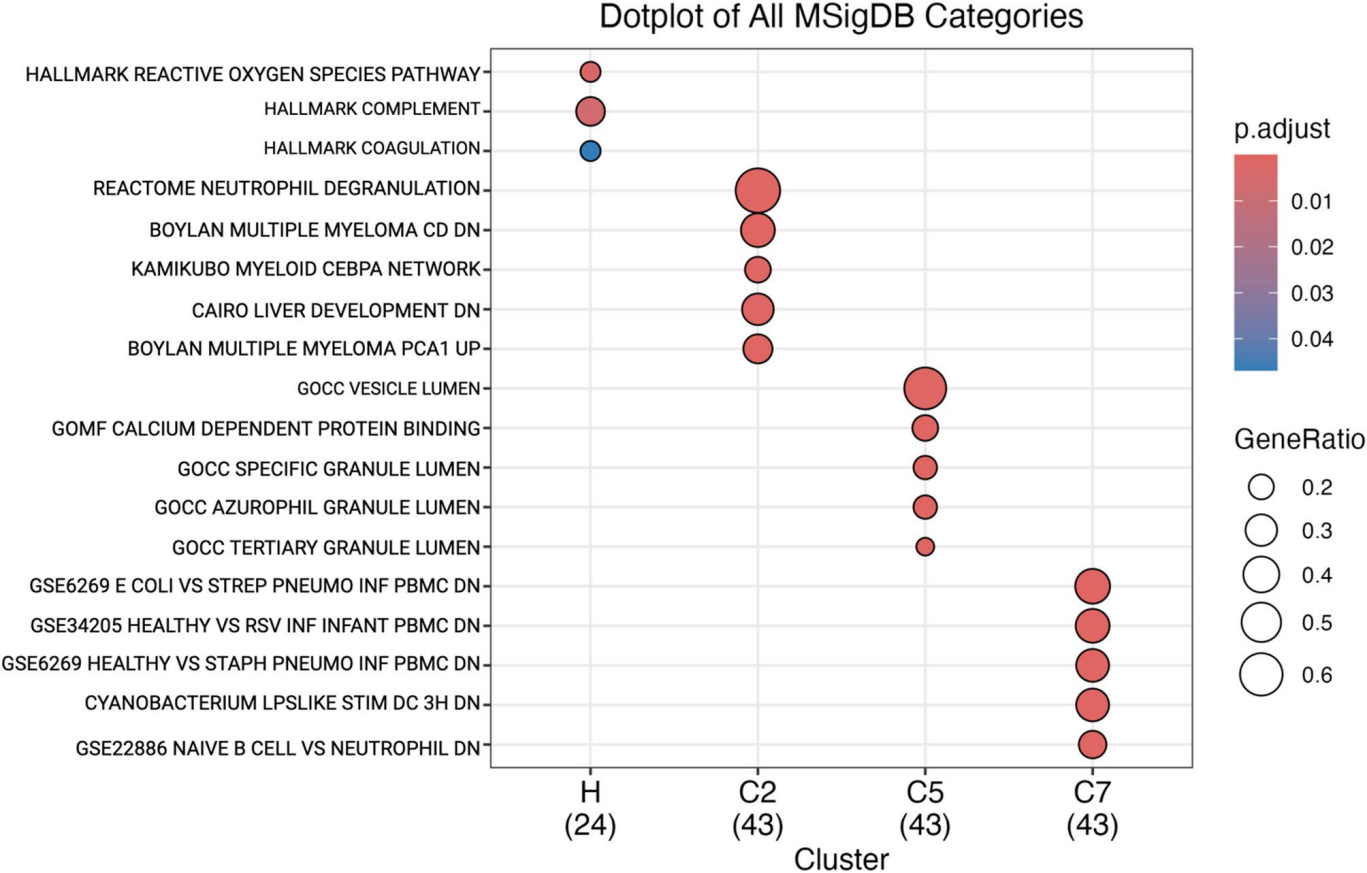
Functional profiling of naturally emerging immunoproteomic CCP classes. The 43 differentially expressed proteins driving differences among CCP classes were evaluated to explore physiologically coordinated behaviors among functionally related proteins to explore the biological meaning that may be attributed to CCP classes. Gene set enrichment analysis was completed with the ClusterProfiler software and molecular signatures database (MSigDB). The legends above include (1) GeneRatio: representing the proportion of input genes associated with a specific functional term or pathway; and (2) p.adjust: representing the adjusted P value significance metric after controlling for multiple hypothesis testing. As per developer guidelines, only the gene sets relevant to the experimental design were included in the reference database via MSigDB gene sets: H: hallmark, C2: curated gene sets, C5: ontology gene sets, and C7: immunologic gene sets. Among the curated gene sets, neutrophil degranulation emerged as the most enriched pathway with (gene ratio: 29/43, adjusted P value = 1.49 × 10^− 35^). With other notable pathways including GOCC vesicle lumen (gene ratio: 328/19525, adjusted P value = 1.34 × 10^− 30^), and GSE4748 LPS related pathway (gene ratio: 14/43, adjusted P value = 5.06 × 10^− 16^). Importantly, the genes detected in the GSE4748 LPS related pathway overlapped completely with a subset of genes from the neutrophil degranulation pathway (see Supplementary Information S11 and S12 for additional details).

**Fig. 4:**
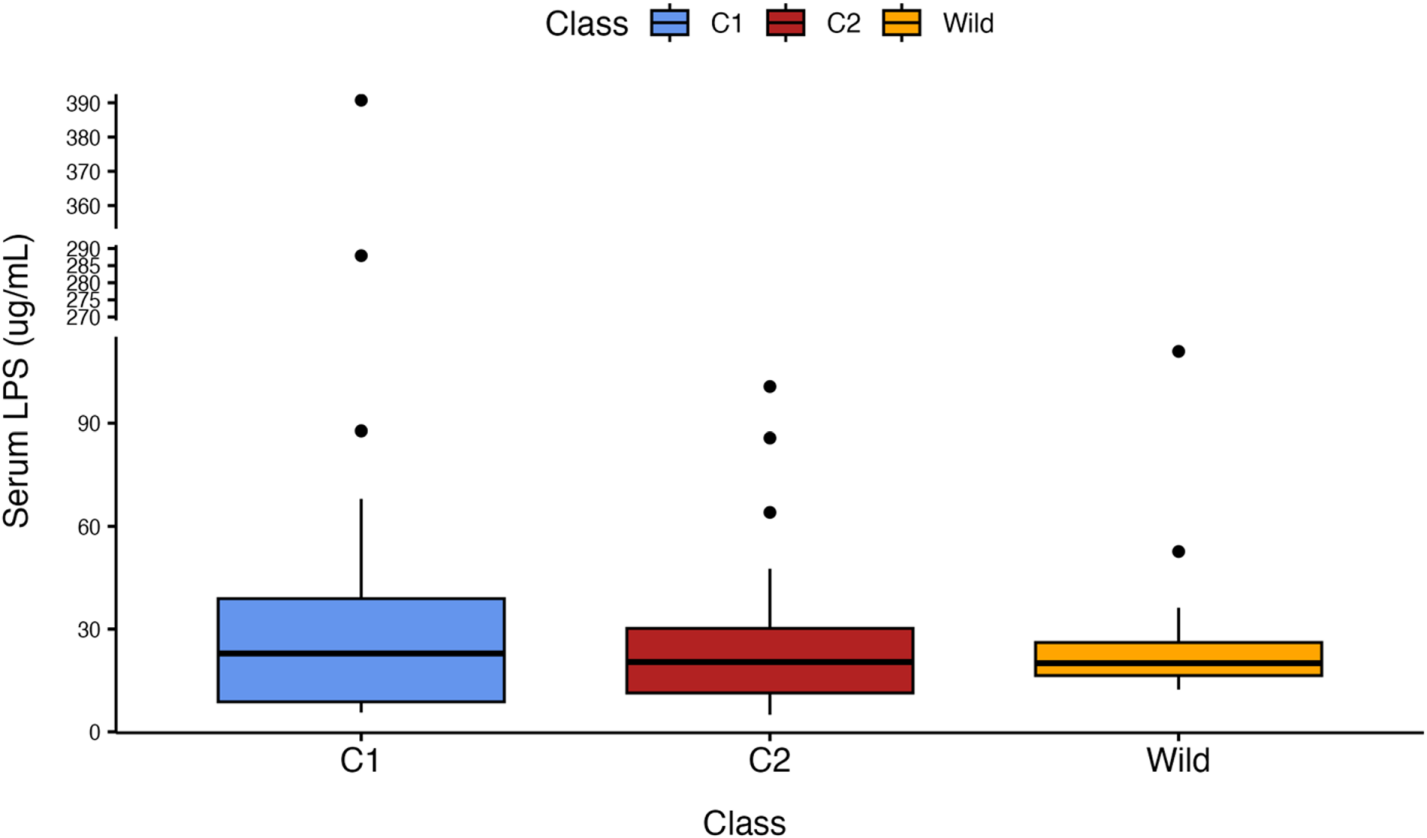
Serum LPS concentrations do not differ between CCP classes 1 and 2 and in situ (wild) black rhinos. X-axis represents classes: CCP classes “C1” and “C2”, and “Wild” controls. No significant differences in serum LPS concentrations were identified by origin (in situ vs. ex situ) or class via linear mixed effects model (Supplemental Information S4 & S13).

Significant differentially expressed proteins were mapped to gene networks to assess biological differences in altered physiological mechanisms (Fig. 3). CCP class 2 was designated as the putative “diseased” group, based on the detection of forty-three candidate inflammatory biomarkers (Fig. 2, Supplemental Information S9) whose biochemical characteristics - including matched proteins and direction of expression - recapitulated proteomic signatures observed in humans and murine model species with related disease phenotypes such as dental/periodontal disease, gastrointestinal dysbiosis, and systemic inflammation (Supplemental information S12A-E). An extensive list of references outlining existing research on proteomic signatures of humans/model species with related disease phenotypes can be found in the supplemental information (Supplemental Information S12E). In the present study, we explored the biological meaning of CCP classes by evaluating coordinated behaviors among functionally related proteins, which was accomplished by profiling the physiological pathways associated with expression of the forty-three candidate inflammatory biomarkers. We identified multiple significant pathways determined via biological term classification and enrichment analysis of gene clusters (Fig. 3; Supplemental Information S11A). This approach allowed for grouping of biological processes and prediction of proteins in co-expression with known functions. Significant pathways of interest included neutrophil degranulation, the complement system, vesicle lumen (gene ontology cell compartment), bacterial lipopolysaccharide (LPS) related pathways, reactive oxygen species, and pathways involved in modulating liver development, coagulation, and calcium dependent protein binding among others (Fig. 3; Supplemental Information S11A-B). Neutrophil degranulation was the most significantly enriched pathway among the ex situ population CCP class comparison. Neutrophil degranulation was detected via differential enrichment of 29 proteins (gene ratio 29/43) including S100A8, S100A9, CD177, S100A12, PGLYRP1, OLFM4, CAMP, MMP9, PRTN3, RETN, S100A11, LTF, LCN2, PYGL, ELANE, ORM2, LTA4H, SERPINB1, GCA, GSTP1, HK3, LGAL23, CAT, MPO, S100P, HBB, MVP, AZU1, and ANZA2. Notably, most (12 of 14) of the proteins detected for the LPS related pathway overlapped with a subset of proteins from the neutrophil degranulation pathway. This extensive overlap, together with additional experimental data (Fig. 4), suggests that the apparent enrichment of the LPS related pathway reflects annotation redundancy rather than a distinct biological pathway (Supplementary Information S11B).

### LPS enzyme immunoassay

After detecting enriched LPS related pathways in CCP class 2, we measured serum LPS values in a subset of samples collected at the same time as *ex situ* PBMCs. Further, we included several samples from in situ (wild) animals that served as a putative “healthy” control (see Supplementary Methods for details). Unfortunately, corresponding PBMC samples from the in situ animals were not available. Serum samples were collected from 27 *ex situ* animals and 19 *in situ* animals. We evaluated ex situ and in situ samples to search for differences in circulating serum LPS concentrations between three groups: CCP class 1, CCP class 2, and in situ animals (Fig. 4). Each ex situ serum sample collected corresponded with a PBMC sample included in the proteomic analyses as these serum samples were collected from the same animals at the same time. Longitudinal samples were obtained from 23/27 ex situ animals and single samples were obtained from 4 ex situ and 19 in situ animals (Supplemental Information S13A). Most longitudinal ex situ serum samples selected reflected instances of CCP class switching. Of 77 total serum samples evaluated, serum LPS concentrations ranged from 4.98 to 390.74 (µg/mL) with a median of 20.70 and mean of 35.26 (µg/mL). A subset of paired samples from 19 ex situ individuals that switched between classes C1 and C2 longitudinally were evaluated; Wilcoxon signed-rank test showed no significant differences in serum LPS concentrations between C1 and C2 (*n* = 15 pairs, V = 97, P value = 0.95) during class switching events.

### Similarities in protein expression patterns related to human disease phenotypes

Differential expression between CCP classes 1 and 2 revealed class 2 samples expressed disease signatures previously reported in humans with periodontitis, gastrointestinal dysbiosis, and systemic inflammation (Supplemental Information S12A-E). Therefore, after generating functional profiles with ClusterProfiler^38^ and MSigDB^39^, we constructed heatmaps of proteins identified as differentially expressed in CCP class 2. The following proteins identified in CCP class 2 with known roles in periodontitis include: S100A8 and S100A9^40^, CD177^41^, PGLYRP1^42,43^, S100A12^44^, IGLL5^45^, MMP9^46^, LTA4H^47^, SERPINB1^48^, CAMP^49^, PRTN3^50^, HPR^51^, S100A11^52^, RETN^53^, LGALS3^54^, IGKC^55^, CAT^56^, LTF^57^, and TXN^58^. Proteins with known roles in gastrointestinal dysbiosis included: S100A8^59^, S100A9^60^, CD177^41^, OLFM4^61^, PGLYRP1^62^, S100A12^63^, IGLL5^64^, MMP9^65^, LTA4H^66^, SERPINB1^67^, ORM2^68^, GCA^69^, CAMP^70^, PRTN3^71^, S100A11^72^, RETN^73^, HBB^74^, LGALS3^75^, IGKC^55^, HK3^76^, A2M^77^, ANXA1^78^, CAT^79^, SRI^80^, LTF^81^, and TXN^82^. Finally, proteins with known roles in the systemic inflammatory process included: S100A8^83^, S100A9^84^, CD177^85^, PGLYRP1^86^, S100A12^87^, OLFM4^88^, LTA4H^89^, SERPINB1^90^, ORM2^91^, GCA^69^, CAMP^92^, PRTN3^93^, S100A11^94^, GSTP1^95^, RETN^96^, LGALS3^97^, IGKC^98^, HK3^99^, A2M^100^, ANXA1^101^, CAT^102^, SRI^103^, LTF^81^, TXN^104^, NCF2^105^, and PYGL^106^. Overall, our findings implicate the oral-gut-liver axis in inflammatory diseases of ex situ black rhinoceros.

## Discussion

We characterized, for the first time, the immune cell proteome (immunoproteome) of any endangered mammal species and identified 1,311 proteins in total. Surprisingly, the immunoproteome when annotated by metadata (subspecies, sex, survey-reported inflammatory phenotype) showed no differences in protein abundance in comparisons between subspecies (eastern vs. southern), sex (male vs. female), or health status (presumed healthy vs. inflammatory phenotype based on reported clinical designations). This prompted further investigation via use of an unsupervised class discovery algorithm (consensus clustering) to identify naturally emerging groups within the dataset. Two separate CCP classes emerged based on biochemical features of individual samples, driven by the differential expression of 43 proteins, which we identified as candidate biomarkers associated with inflammatory pathways. When these proteins were examined for coordinated activity within related functional pathways, neutrophil degranulation emerged as the most prominent signature and implicated CCP class 2 as the putative inflammatory group. Pending further validation, the 43 candidate inflammatory biomarkers identified in this study may advance understanding of the underlying disease mechanisms and support the future development of diagnostic and therapeutic tools for managing inflammatory disease in this species. Overall, molecular features of CCP class 2 recapitulate disease signatures previously reported in humans with periodontitis, gastrointestinal dysbiosis, and systemic inflammation. Results of this study implicate for the first time that inflammatory disease processes in black rhinos are modulated by an overarching oral-gut-liver axis dysregulation.

Neutrophils are the most abundant innate immune cells in circulation (black rhino reference range 5.24 × 10^3^/mL)^107^ and the first to respond to microbial infections, tissue injury, and inflammation. Neutrophils display diverse physiological actions in immune function through phagocytosis, NETosis (cell death related to neutrophil extracellular traps), and degranulation^108^. Neutrophil degranulation accompanies acute inflammatory responses linked to microbial invasion and phagocytosis, inflammation, or tissue injury^109^. Chemically, this defensive reaction occurs through the release of hydrolytic enzymes when neutrophils produce intracellular vesicles, “granules”, that contain proteases. This process can occur in both the intra- and extracellular space^110^. Extracellular neutrophil degranulation is necessary for neutralizing invading microbes. However, excessive neutrophil degranulation can injure host tissue^111^. Some microbial pathogens can even distort protective effects of neutrophil degranulation as a virulence strategy by dysregulating or inducing excessive neutrophil degranulation^110^. In the present study, we identified neutrophil degranulation as the predominant physiological pathway that drives separation of CCP classes based on biochemical features of individual samples. Of the 29 proteins detected in the neutrophil degranulation pathway (S100A8, S100A9, CD177, S100A12, PGLYRP1, OLFM4, CAMP, MMP9, PRTN3, RETN, S100A11, LTF, LCN2, PYGL, ELANE, ORM2, LTA4H, SERPINB1, GCA, GSTP1, HK3, LGAL23, CAT, MPO, S100P, HBB, MVP, AZU1, and ANZA2), four encode for neutrophil granules: primary (PRTN3), secondary (CAMP, LTF), and tertiary (MMP9) neutrophil granules. Neutrophil degranulation across the oral-gut-liver axis likely contributes to the development of commonly reported dental, gastrointestinal, and liver disease phenotypes in black rhinos.

Black rhinos in human care experience dental disease more often than their wild counterparts^112^. The origins of dental/periodontal disease are not fully understood, though it is plausible that microbes enter circulation via damaged periodontal tissue and stimulate an inflammatory response^113^. In the current study, housing institutions reported that nearly one-third of ex situ black rhinos showed clinical signs of dental/periodontal disease. However, the true proportion of animals with dental/periodontal disease is unknown as detailed dental examinations require immobilization and are not routinely performed on every animal. Black rhinos are specialized browsers that evolved low-crowned teeth to consume highly fibrous plants such as tree branches and shrubs. In comparison, rhinos in human care eat a diet comprised of hay, commercially available specialized concentrate feeds, and browse as available. Previous research indicates that feeding browsers plant species containing high silica concentrations (hay) may contribute to dental diseases^114^. As a result, an enormous opportunity exists for future research to utilize controlled feeding trials to assess relationships that may exist between diet and dental health of black rhinos. The proteins detected in this study have relevance in identifying periodontitis in human patients. In humans, calprotectin (S100A8/A9) levels are associated with periodontitis disease severity^115^. Here, we detected increased expression of granule protein olfactomedin 4 (OLFM4) in CCP class 2. Expression of OLFM4 is significantly upregulated in human patients during sepsis and could itself be pathogenic during severe infection^116^. We also detected increased expression of glycoprotein CD177 in CCP class 2, which may imply the involvement of microbe-driven inflammation. Overexpression of CD177 has been previously linked to microbe-triggered neutrophil migration with CD177 + neutrophil subsets preferentially recruited to the gingival crevice of human patients with periodontitis^41^. Neutrophil presence increases in tissues affected by periodontitis; with neutrophils exhibiting a proinflammatory phenotype^117^.

Gastrointestinal dysbiosis is commonly reported in ex situ black rhinos and includes chronic intermittent diarrhea^6^ and enteritis of infectious or unknown etiology^106^. Previous research evaluated the fecal gut microbiomes of in situ and ex situ black rhinoceros and demonstrated that gut microbiomes are highly divergent between these two populations^119^. Specifically, gut microbiomes of animals in human care resembled domesticated ruminant guts and suggests unknown microbes of wild rhinos are being replaced by microbes found in conventional domesticated livestock^119^. Authors postulated that dietary differences among ex situ and in situ animals likely contributed to alterations in microbiome composition^119^. Although the origins of gastrointestinal dysbiosis phenotypes are not fully understood in black rhinos, the results of the present study mirror findings reported in human patients with gastrointestinal dysbiosis where neutrophils express elevated CD177 and S100A12 in patients with ulcerative colitis^120^ and PGLYRP1 in patients with irritable bowel disease (IBD)^121^. In states of gastrointestinal dysbiosis, gut barrier dysfunction can occur resulting in microbial translocation - the movement of microbes and their metabolites from the intestinal lumen to the hepatic portal vein^122^. This can result in a pro-inflammatory response and increased intestinal membrane permeability, which has been previously implicated in the influx of pathogen-associated molecular patterns that prime hepatic inflammation^123^. Thus, the connection between gastrointestinal dysbiosis and hepatic inflammation warrants further studies in black rhino disease syndromes.

The liver is strategically located between the gastrointestinal tract and circulatory system and is regularly exposed to bacterial products, toxins, and food-derived antigens^124^. Liver disease, specifically iron overload disorder (IOD), is a documented condition of ex situ black rhinos^7,19,125–129^. A retrospective (1995–2022) pathology review of black rhinos housed in European facilities identified IOD was nearly ubiquitous across animals^8^. Hepatic infiltration of neutrophils is commonly observed in many types of liver diseases, though an overwhelming activation of neutrophils can induce hepatocyte injury by producing reactive oxygen species (ROS), degranulation, inflammatory, and immune mediation^130^. Systemic microbial invasion drives S100 protein recruitment for immune cell activation. A member of the S100 family, calprotectin (S100A8/A9), was the most differentially expressed protein in this study. Calprotectin is known to be a metal-sequestering host defense protein that reduces bacterial pathogen virulence via iron sequestration^131^. This finding mirrors results reported in mouse models of acute and chronic liver injury, which identified the toll like receptor 2 (TLR2) and calprotectin (S100A8/A9) signaling pathways as key regulators of hepatic chemokine ligand 2 (CXCL2) and tumor necrosis factor (TNF) expression and subsequently, neutrophil infiltration^132^. Further, we detected increased expression of matrix metalloproteinase-9 (MMP-9) in CCP class 2. In humans and murine model species with liver ischemia-reperfusion (IR), upregulation of MMP-9 is accompanied by massive neutrophil infiltration, increased levels of proinflammatory cytokines, and impaired liver function^133^. Similarly, cyclooxygenase-2 (COX-2) derived from hepatocytes reduces liver injury by decreasing endoplasmic reticulum stress, neutrophil infiltration, and oxidative stress and escalates autophagy and apoptosis^134^. We previously detected upregulated prostaglandin E1 (PGE1), a metabolic byproduct of the COX-2 component of the arachidonic acid pathway, in the present rhino population when comparing animals with and without clinical signs of inflammatory phenotypes^9^.

Although we identified differential expression of calprotectin and neutrophil granules, we did not find differences in serum LPS within our study population. Blood LPS concentrations (1.9 to 20 pg/mL) have been reported in healthy humans^135^. In a severe case of sepsis in humans, LPS levels increased to 580 pg/mL^136^. In horses with abdominal disease, plasma LPS concentrations ranged from 25.5 to 93 pg/mL^137^. In the present study, black rhino serum LPS levels ranged from 4.98 to 390.74 µg/mL (4.98 × 10^6^ to 3.9 × 10^8^ pg/mL) and significant differences in LPS levels could not be detected by CCP class. In our study, serum LPS levels in this study were similar across rhino samples regardless of CCP class, sex, subspecies, health status, or origin (in situ or ex situ). Overall, LPS values for the black rhinos, including in situ animals, were substantially higher than ranges reported for humans^135^ and the horse^137^. The physiological significance of these high values remains unclear, and more research is needed to understand why serum LPS concentrations are remarkably elevated in black rhinos compared to other species.

In model species, in vitro experiments evaluating neutrophil migratory decision making for high, intermediate, and low dose LPS demonstrated that even very low levels of LPS can significantly dysregulate neutrophil migratory phenotypes^138^. However, the mechanism through which LPS may influence neutrophil function remains unclear. In a murine model of acute lung injury exploring whether LPS could induce NET release in in vivo and in vitro contexts, researchers determined LPS alone could not induce NET release during direct contact with neutrophils; however, LPS-activated platelets could elicit NET generation^139^. In the present study, although we detected enrichment of an LPS related pathway, our analysis indicated that this signature likely reflects an annotation redundancy or general neutrophil activation signature, rather than an LPS-driven immune response, and majority of the genes mapping to the LPS related pathway overlapped with a more significant and prominent pathway - neutrophil degranulation. Taken together with the circulating serum LPS values that showed high concentrations across all black rhinos evaluated, with no significant differences within the cohort, it is possible that other microbial products (bacterial DNA, peptidoglycan, lipoteichoic acid, flagellin, ribosomal DNA, or unmethylated CpG-containing DNA) may drive microbial translocation^140^. For instance, *Aspergillus fumigatus*, a pathogenic fungus in immunocompromised humans, can produce toxins including fumagillin, which in vitro has been shown to inhibit normal neutrophil function and disrupt degranulation; and in vivo, appears to facilitate colonization by other microbial taxa leading to systemic disruption of the host immune response^141^. Collectively, these findings highlight the critical need for future research to understand how a broader spectrum of microbes and their products-including those from gram-positive bacteria, archaea, viruses, fungi, or protists- may initiate inflammatory responses through neutrophil degranulation in black rhinoceros.

**Fig. 5:**
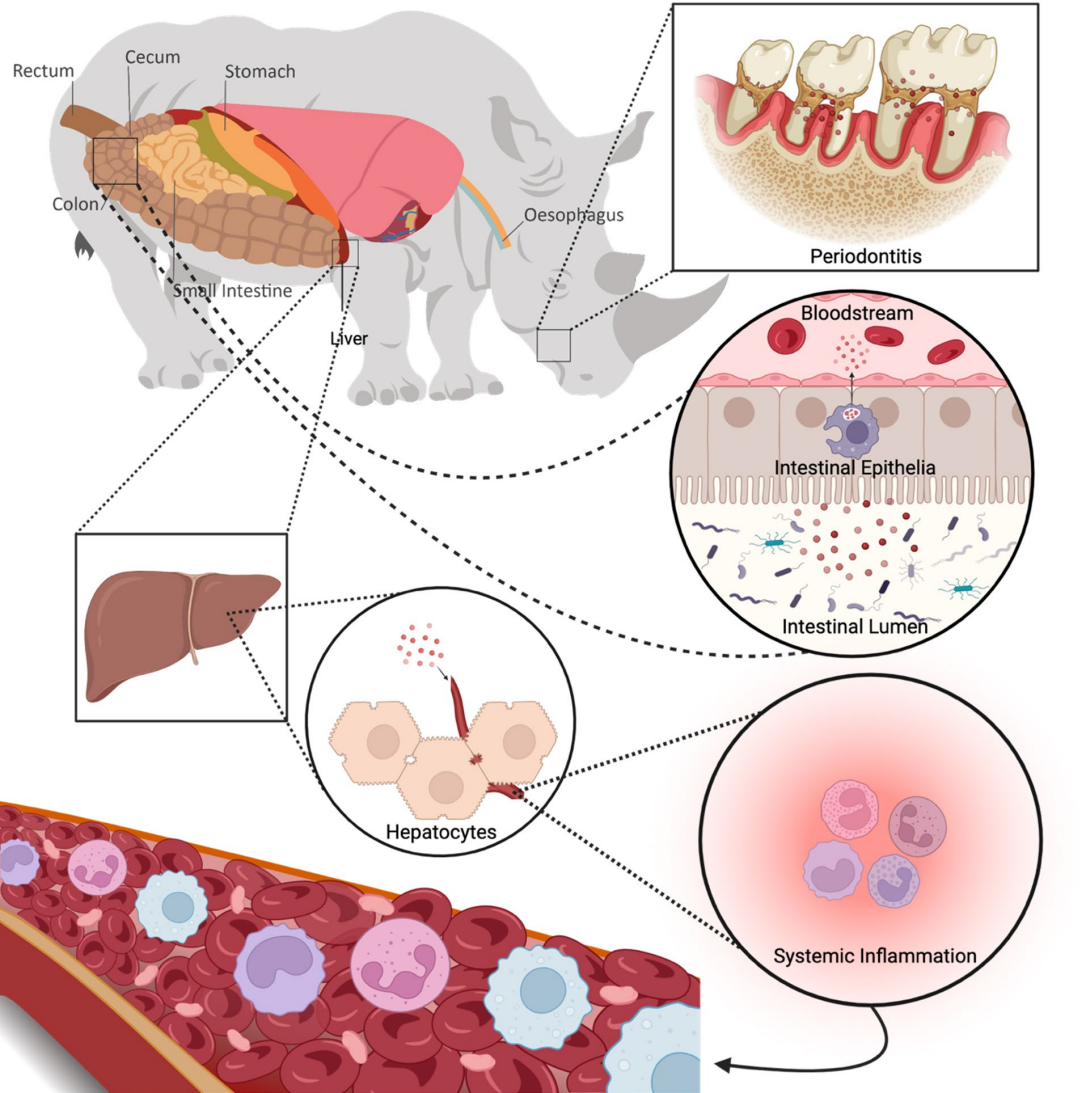
The oral-gut-liver axis modulates disease syndromes in ex situ black rhinoceros. Here we propose a mechanism through which inflammatory microbial products reach the liver via the hepatic portal vein and stimulate the immune response associated with ex situ inflammatory phenotypes (periodontitis, gastrointestinal dysbiosis, and systemic inflammation). This figure was created in BioRender and the rhinoceros graphic was created by Ms. Sabrina Amann-Ross at CorCom Inc.

Here we propose a mechanism through which inflammatory microbial products reach the liver via the hepatic portal vein and stimulate the immune response associated with ex situ inflammatory phenotypes (periodontitis, gastrointestinal dysbiosis, and systemic inflammation) (Fig. 5).

We postulate that diverse neutrophil functions modulate the immune response across the oral-gut-liver axis during microbial infections/translocation, periods of prolonged inflammation, and massive chronic neutrophil infiltration. As the liver is the key organ that responds to systemic inflammation, our data suggests that oral-gut-liver axis is impacted in *ex situ* black rhinos affected by various disease syndromes (Fig. 5).

### Study limitations and strengths

These data provide novel insights into the molecular underpinnings of inflammatory disease in the critically endangered black rhinoceros. This research was conducted in the absence of controlled laboratory conditions, as conducting biomedical research on endangered species is challenging for several reasons: (1) as an endangered species, there are a limited number of animals in human care (ex situ) available for systematic research, (2) even animals in the wild may not be truly healthy due to stress, poaching pressures, and food availability, (3) it is not possible (or ethical) to house large groups of rhinos under laboratory conditions; (4) animals live (in situ and ex situ) in diverse locations around the world, and dietary/environmental variations exists, and (5) in some cases, immediate medical interventions are necessary to ensure the health and welfare of these animals, especially those in human care. Consequently, the lack of true control “healthy” animals and the necessity to actively manage animal health in human care often confound research outcomes. In the present scenario, we are left with two options: (1) the option to do nothing to assess population-level ex situ animal health due to unavailable controlled conditions, or (2) the option to work within existing constraints to develop novel ways of assessing population-level ex situ animal health. Ethically, the latter is the best option. Animals in zoological facilities and breeding centers serve as a valuable resource for understanding physiological mechanisms of wildlife species. Maintaining healthy genetically viable animals in human care can reduce extinction risks. Perhaps the extinction of the western black rhinoceros subspecies^5^ could have been prevented if healthy ex situ populations were available for reintroduction efforts. With novel approaches to understand and enhance animal health and wellbeing, the future of the eastern and southern black rhinoceros subspecies may be bright.

In the present study we elected to use data-driven unsupervised machine learning to identify naturally emerging groups within this novel immunoproteomic dataset and generated new knowledge on disease mechanisms in black rhinos. These findings may prove useful in determining drivers of ex situ disease syndromes so long as the possibility of confounding factors is considered when interpreting results and thorough secondary validation studies are conducted. When conducted, these studies would have to rely on immunologic tools (antibodies) commercially available for domestic species and humans. But there is no guarantee that these tools would identify rhino antigens. Despite these potential limitations, our approach demonstrates that enormous value exists in identifying molecular pathways and/or candidate biomarkers that may possess clinical utility (to differentiate between subclinical and clinical disease states) in studying rare diseases or disease syndromes of rare species.

## Conclusions and future directions

We propose that neutrophil degranulation drives immune dysfunction throughout the oral-gut-liver axis. In our proposed mechanism, we postulate that microbial translocation activates neutrophil degranulation; whereby microbial products, endotoxins, or other inflammatory products reach the liver via the hepatic portal vein and stimulate a systemic immune response resulting in periodontitis, gastrointestinal dysbiosis, and/or systemic inflammation. In humans with gastrointestinal dysbiosis, neutrophil infiltration into the intestinal mucosa is a hallmark of active IBD, with calprotectin and other neutrophil granule proteins as key biomarkers^142^. Our data mirrors these findings with calprotectin being the most differentially expressed protein between CCP classes. Future research priorities include evaluation of (1) the role of neutrophil degranulation (and related processes: NETosis, oxidative stress, and potential microbial invasion) in inflammatory disease syndromes of black rhinoceros, (2) the validity of the candidate biomarkers identified in the current study for differentiating between inflammatory disease states in black rhinoceros, (3) the influence of diet including seasonal variation (browse quantity & quality) on black rhino metabolic and immune health, and (4) the involvement of dietary changes and veterinary interventions in class switching. Specifically, this should involve the characterization of molecular features associated with inflammation in distinct immune cell populations and clinical phenotypes of ex situ black rhinos. Studies should also evaluate these molecular features in the context of standard clinical biomarkers of inflammation including serum amyloid A (SAA), haptoglobin, and immunoglobulins. Ideally, future works should identify the mechanistic underpinnings of microbial translocation across the intestinal or periodontal membrane, neutrophil infiltration, and subsequent liver inflammation in this species.

## Methods

### Sample acquisition & preparation

Ex situ samples were acquired in accordance with Smithsonian Institution International Animal Care and Use (IACUC; Protocol #19–28). In situ samples included in the LPS experiment were acquired in accordance with the Convention on International Trade in Endangered Species (CITES) of wild fauna and flora import permit (#23US35783E/9), export permit (#318807), threatened or protected species permit (#65762), Sect. 20 permit (South Africa; #12/11/1/7/2–1706JD), and biomaterials transfer agreement with South African National Parks (#BMTA001/23).

Eighty EDTA whole blood samples were collected (BD vacutainer EDTA tubes) from 27 black rhinos and shipped cold (4 °C) overnight to the Smithsonian’s National Zoo & Conservation Biology Institute in Front Royal, Virginia, USA. PBMCs were isolated from whole blood using a Ficoll density gradient and series of washing steps^143^. Resulting cell pellets (PBMCs) were stored frozen at −80 °C until mass spectrometric analyses. Cell pellets (PBMCs) were lysed with 50 µL 4 M urea. Protein concentration was determined with the Pierce Coomassie Protein Quantification kit using the Eppendorf BioSpectrometer Basic (Eppendorf AG, Germany), as per manufacturer’s instructions. Protein concentrations were then normalized to ensure the same amount of protein per sample (20 µg protein/40 µL sample in 4 M urea solution) before digestion. Samples were reduced with 1 M dithiothreitol (DTT), alkylated with 6 µL iodoacetamide solution in ammonium bicarbonate (057K53011 Sigma-Aldrich, Burlington, MA) and digested with 34 µL master mix (33 µL 500 mM ammonium bicarbonate and 1 µL 0.5 µg/µL sequencing grade modified trypsin (REF: V5117, Promega Corporation, Madison, WI). The trypsin digestion occurred at 37 °C for four hours. Digested proteins were purified using ZipTip™ pipet tips and washed with two buffers, buffer A: 0.1% formic acid and buffer B: 80% acetyl nitrile in a specific sequence to elute washed peptides (Supplementary Methods S13). Samples were then dried using a Savant™ Universal SpeedVac™ system UVS800DDA (Thermo Fisher Scientific, Waltham, MA) until dry (20–25 min). After drying, samples were stored at −20 °C until the Orbitrap Exploris™ mass spectrometer run (CAT: BRE725539, Thermo Fisher Scientific, Waltham, MA).

### Mass spectrometry

Samples were resuspended in 10 µL buffer A and the suspension was loaded into mass spectral tubes. Tryptic digests were analyzed in an Exploris 480 Mass Spectrometer (data dependent acquisition) at the Center for Applied Proteomics and Molecular Medicine at George Mason University (Supplementary Methods). Mass spectral output files were saved as Thermo.RAW files for each PBMC sample analyzed.

### Lipopolysaccharide enzyme-linked immunosorbent assay

LPS values were measured in 77 serum samples corresponding with three groups (1) CCP class 1 (*n* = 34), (2) CCP class 2 (*n* = 24), and (3) wild animals (*n* = 19), using manufacturer protocols and a commercially available competitive LPS ELISA kit (UNFI0091; AssayGenie, Dublin, Ireland). To ensure assay efficacy, a parallelism was run, and standard curve obtained (Supplementary Methods). Ex situ serum samples corresponded with PBMC samples (CCP classes 1 and 2) as they were collected from the same animals at the same time points as PBMCs.

## Computational methods

### Reference database

Using a recently generated platinum-quality genome assembly for the southern black rhino (trio-binning method), a new black rhino reference proteome database^144^ was generated by the National Center for Biotechnology Information using standard annotation pipelines. This rhino-specific proteome was queried to identify the peptides using MetaMorpheus (v 1.0.5)^145,146^. A total of 4,007 proteins were identified (FDR < 1%) and included in downstream analyses. We then used PAW BLASTer^147^, a tool for converting peptide identifications (IDs) from understudied species to homologous IDs in better characterized species (humans and model organisms). We converted black rhino gene and protein IDs to homologous human gene and protein IDs^148^ to ensure compatibility with functional pathway analysis software downstream.

### Imputation and missing values filtering

The remaining computational analyses were conducted using R/RStudio (version 4.4.2). With a data set constructed from the remaining 4,007 proteins (rows representing identified peptides and columns representing samples), we removed proteins with ≥ 25% missing values. After removing missing values and keeping only proteins quantified in at least 75% of samples, 1,326 proteins remained. We then applied the missForest imputation algorithm (R package missForest version 1.4)^149,150^ to impute the residual missing values. The resulting intensity values were log2 transformed before analysis (log2(intensity + 1)). Homologous human gene identifiers representing experimentally detected proteins were assigned with PAW BLASTer (version 1.31.2022) to represent proteins detected in the dataset using the developer’s make_subset_DB_from_list3.py and db_to_db_blatster.py scripts. When multiple peptides/proteins were encoded for by the same gene, we aggregated on the median spectral intensity value for the corresponding gene identifiers, resulting in, 1,311 proteins with unique gene identifiers in the final data used for analyses. Gene identifiers remaining represented genes that encoded for experimentally detected proteins and were suitable for functional profiling downstream.

### Mixed effects modeling

To evaluate if any hardwired biases were present in the dataset due to unevenness in repeated measures sampling, a linear mixed effects model was fit to the data with log2(protein intensities) as the response variable, covariates subspecies, sex, season, health phenotype, and age class as fixed effects; and housing institution, sampling period, and individual animal as random effects. Formula: Intensity ~ Subspecies + Sex + Season + Phenotype + AgeClass + (1 | Institution_ID/BR_ID) + (1 | SamplingPeriod). Graphical diagnostics generated from the model residuals indicated that the assumptions of normality and homoscedasticity were satisfactorily met (Supplemental Information S4).

### Differential expression analysis and consensus clustering

Differential expression of proteins for each of the covariate comparisons (subspecies, sex, and health phenotype) were analyzed using linear models for microarray data, limma^151^ version 3.60.6. Significantly differentially abundant proteins were visualized with a volcano plot using ggplot2 (v. 3.5.2)^152^. We evaluated three unsupervised clustering algorithms (K-means, hierarchical clustering, and consensus clustering) to assess groups that naturally exist within the dataset (Supplemental Information S7). Of these, consensus clustering proved to be the most robust and stable approach for class discovery given its reduced dependence on random starts compared to K-means and hierarchical clustering approaches. ConsensusClusterPlus^36^(CCP) version 1.68.0 was used for unsupervised class discovery where the top 25% most variable proteins within our data was used and k = 2 was determined to be the best fit suggesting the presence of two distinct classes of samples in the dataset (ConsensusClusterPlus(maxK = 4, reps = 1000, seed = 222, pItem = 0.8, pFeature = 1, distance = “pearson”, clusterAlg="hc”)). Parameters for this analysis included an agglomerative hierarchical clustering and Pearson correlation distance method to ensure median centered (median absolute deviation; MAD) protein values. The algorithm was blinded to all covariate (metadata) information while determining (1) the number of groups that potentially occur in the data, and (2) assigned group membership for each sample.

### Functional profiling

The R packages ClusterProfiler (v 4.17.0)^38^ and MSigDB (v 24.1.0)^39^ were used to assess patterns that differed between CCP class 1 and class 2 using 1,000 iterations. As per developer recommendations, gene sets were selected in accordance with our experimental design. H: hallmark, C2: curated gene sets, C5: ontology gene sets, and C7: immunologic gene sets. Heatmaps were generated to visualize the log2 transformed mass spectral intensities of gene IDs that were differentially expressed between CCP classes 1 and 2 using R packages pheatmap^153^ version 1.0.12 and dendextend^154^ version 1.19.0 used for hierarchical clustering of gene IDs based on variance of mass spectral intensities.

## Supplementary Information

Below is the link to the electronic supplementary material.

Supplementary Material 1

## Data Availability

Data and scripts used in this study are available at Smithsonian Institution’s Figshare site: https://figshare.com/s/b49afc55c401b6602358.
