# Supplementary material for: Immunoproteomic insights into inflammatory diseases of the critically endangered black rhinoceros (Diceros bicornis)
Source: Sci Rep. 2026 Mar 14;16:13535. doi: 10.1038/s41598-026-43055-0 (PMC13121450; doi:10.1038/s41598-026-43055-0)
Supplement: Supplementary file 1 — Supplementary Material 1 [file 41598_2026_43055_MOESM1_ESM.docx]

Supplementary Information

**Immunoproteomic insights into inflammatory diseases of the critically endangered black rhinoceros (*Diceros bicornis*)**

**Table of Contents**

**S1: Blank Qualtrics animal health survey**

**S2: *Ex situ* enrollment metadata**

**S3: Sampling periods by individual, season, and number of sampling events**

**S4A: Flowchart for computational pipeline**

**S4B: Screening for potential hardwired biases within the immunoproteomic dataset**

**S5A-G: PCA ordinations of immunoproteome plotted by sample and colored by metadata covariates**

**S6: Differential expression metadata driven comparisons (non-significant)**

**DiffExpressionByMetadata.xlsx** (Subspecies, Sex, Health phenotype, Age class comparisons)

**S7: Serum amyloid A (SAA) and health status**

**S8A-C: PCA ordinations by unsupervised clustering methods**

**S8D:** Alluvial plot of consensus and hierarchical cluster assignments

**S8E:** Testing for significant associations between CCP classes and metadata covariates (not significant).

**S9: Differential expression of proteins driving naturally emerging groups (CCP classes)**

**S10A-C: Evidence for temporal fluctuations in inflammatory phenotypes.**

**S10A:** Class switching table

**S10B:** Class switching Sankey diagram

**S10C:** Class switching network diagram

**S11A: Functional profile data for Figure 3 reflecting biological interpretation of naturally emerging groups (CCP classes).**

**S11B: Evidence for LPS as an annotation redundancy, rather than an LPS-driven immune response.**

**S12: Heatmaps of differential expression from proteins driving differences in naturally emerging groups (CCP classes)**

**S12A:** Heatmap of proteins driving naturally emerging groups (CCP classes).

**S12B:** Heatmap of proteins upregulated in CCP class 2 with known roles in periodontitis.

**S12C:** Heatmap of proteins upregulated in CCP class 2 with known roles in gastrointestinal dysbiosis.

**S12D:** Heatmap of proteins upregulated in CCP class 2 with known roles in systemic inflammation.

**S12E:** Literature Cited list for proteins with known roles in reported disease phenotypes.

**S13: Lipopolysaccharide (LPS) Enzyme Immunoassay (LPS EIA) Parallelism**

**S13A**: LPS experimental design

**S13B:** LPS EIA parallelism plate

**S14: Supplementary Methods**


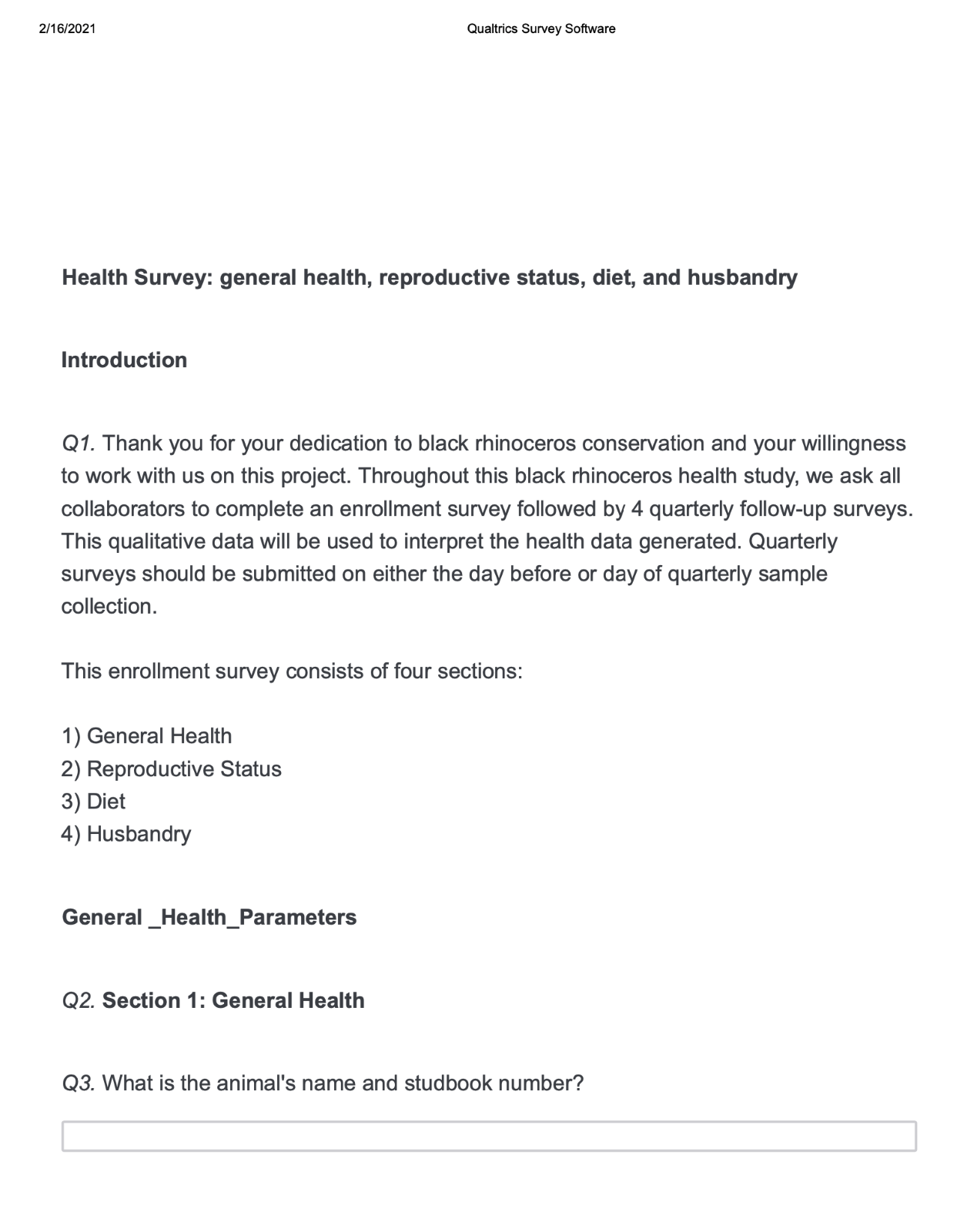


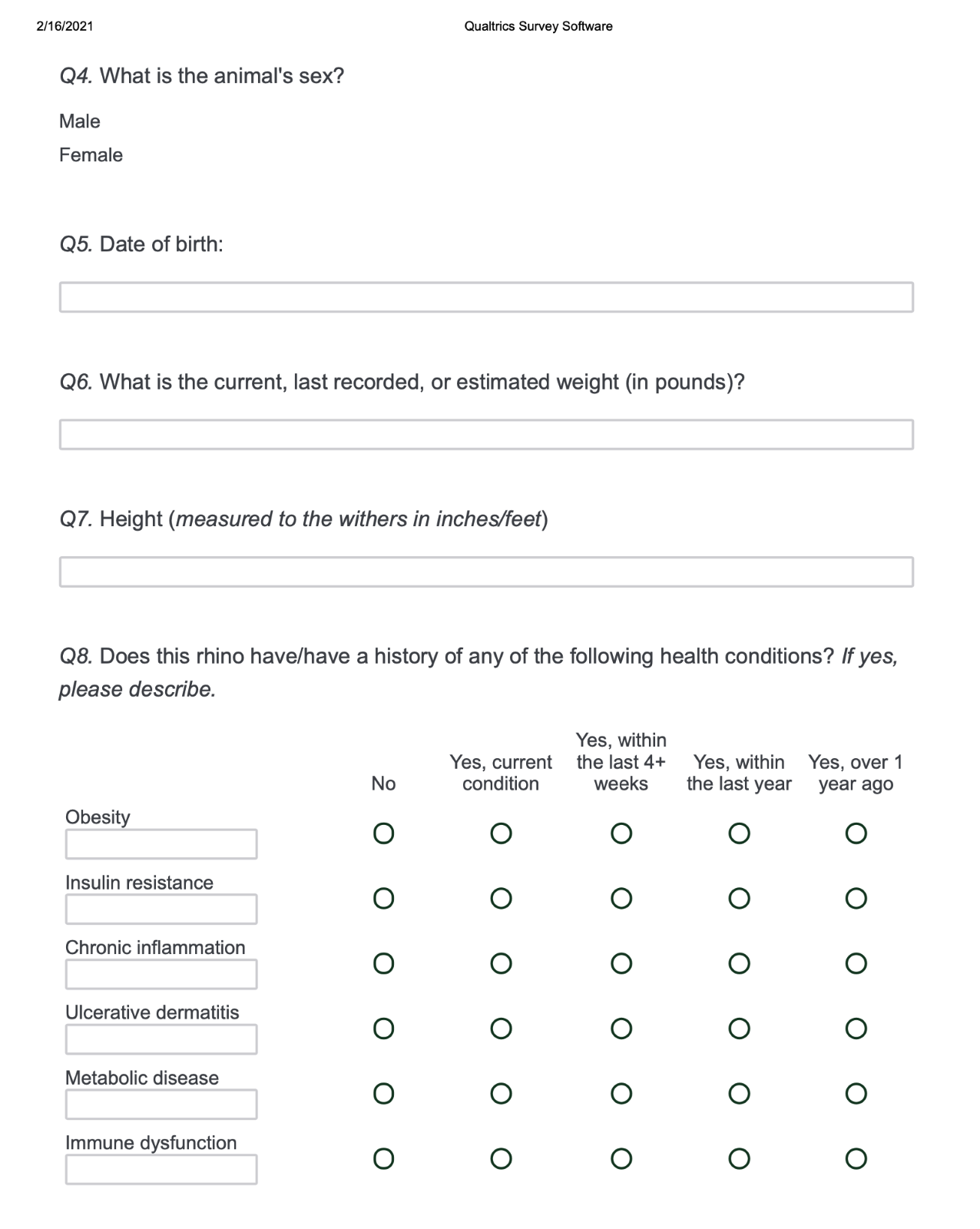


**S1: Blank Qualtrics animal health survey.**

***Survey.*** We surveyed black rhino-housing institutions using the Qualtrics survey software. Information collected included data pertaining to the health and reproductive history, nutrition, and husbandry of each rhino enrolled in this study (S2). This survey data has been previously published (Corder *et al.* 2023) in a related study on the serum metabolome of this same black rhinoceros population.

S2: *Ex situ* enrollment metadata. Disease phenotypes reported by housing institutions (2019-2021) where *n*=number of animals out of 27 individuals with recent or current histories of reported disease phenotypes. Other disease phenotypes reported included past curvularia infections, nail cracks, and mild pododermatitis.


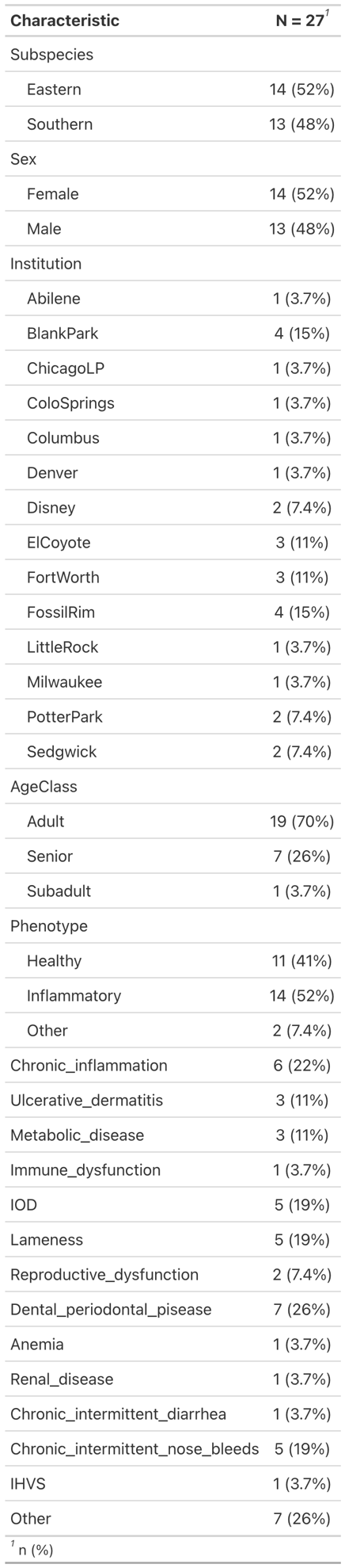

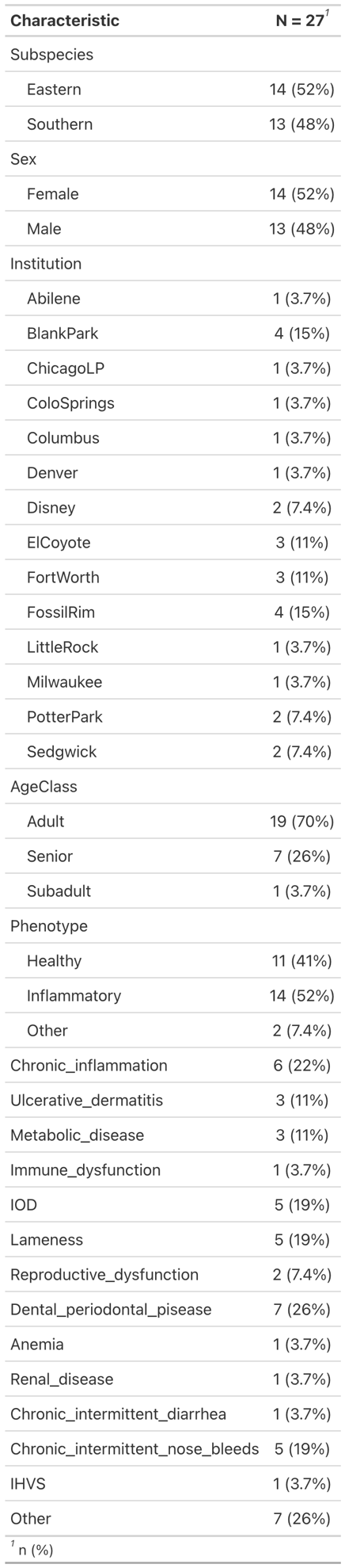


**
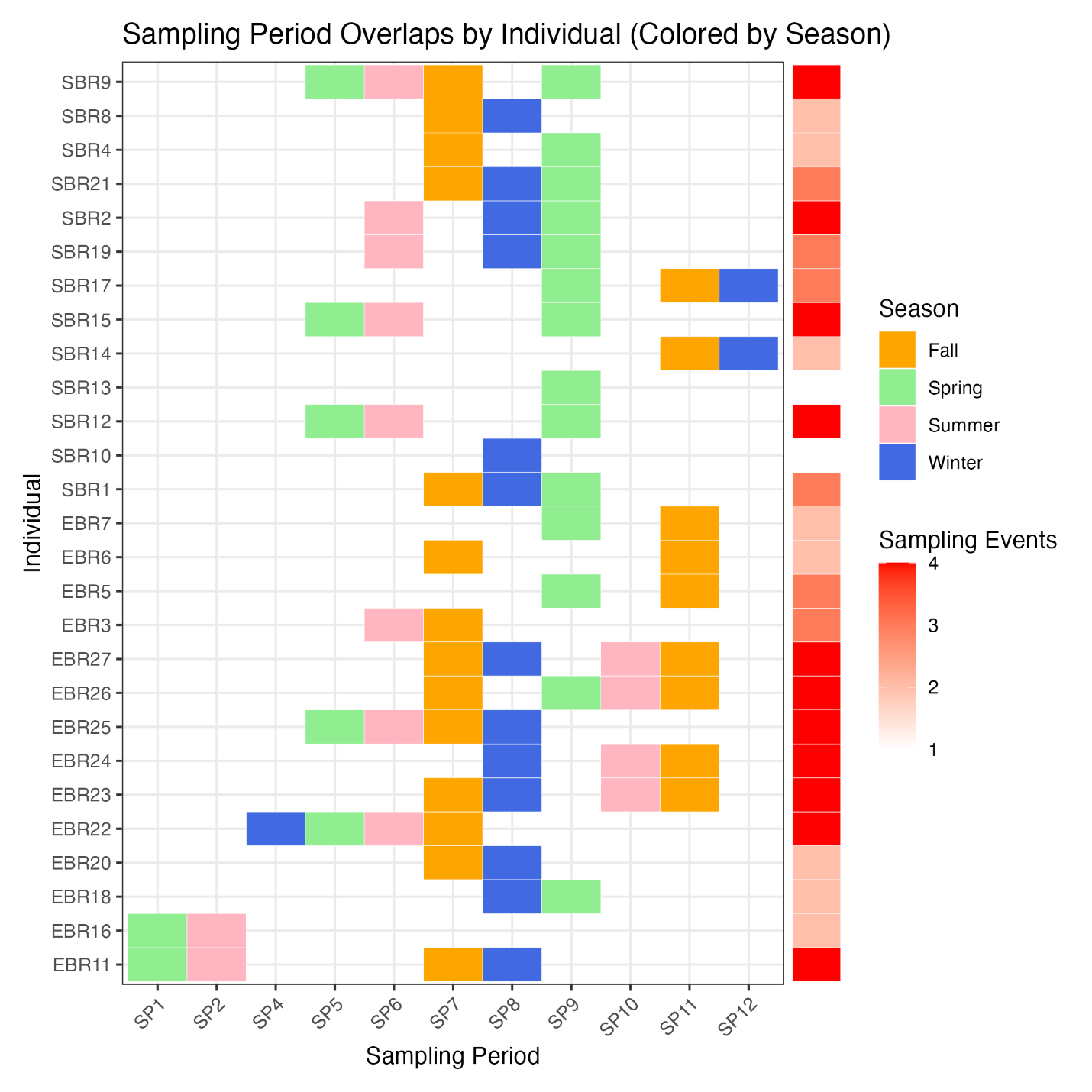
**

**S3: Sampling periods by individual, season, and number of sampling events.**

Sampling occurred among 27 individuals across 12 sampling periods (SP) where each sampling period represented 3 months from spring 2019 to winter 2022. Four seasons included winter: Jan-Mar, spring: Apr-June, summer: July-Sept, fall: Oct-Dec. Number of longitudinal sampling events denoted in white-red legend ranged from 1 (white) to 4 (bright red) longitudinal sampling events. Uniform sampling was not possible given the inherent constraints of working with an endangered species managed at many institutions across the United States during the global pandemic.

**
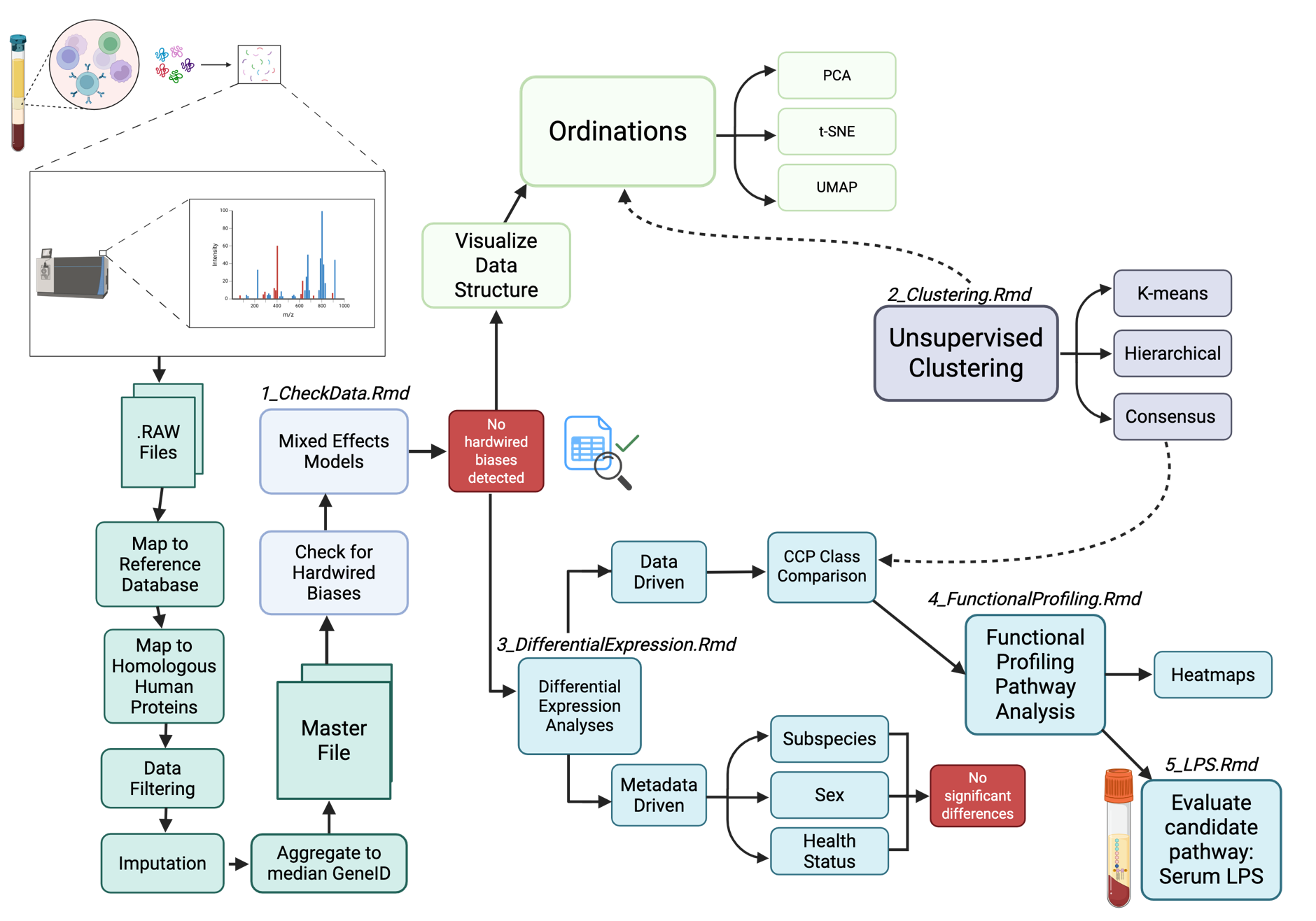
**

**S4A: Flowchart for computational pipeline.** Flowchart depicts computational pipeline from .RAW files through scripts including data quality checks, unsupervised clustering, data visualizations and ordinations, differential expression analyses, functional profiling, and statistics for serum LPS concentration comparisons. Specific scripts for respective analyses are named within the above pipeline including: 1_CheckData.Rmd, 2_Clustering.Rmd, 3_DifferentialExpression.Rmd, 4_FunctionalProfiling.Rmd, and 5_LPS.Rmd.


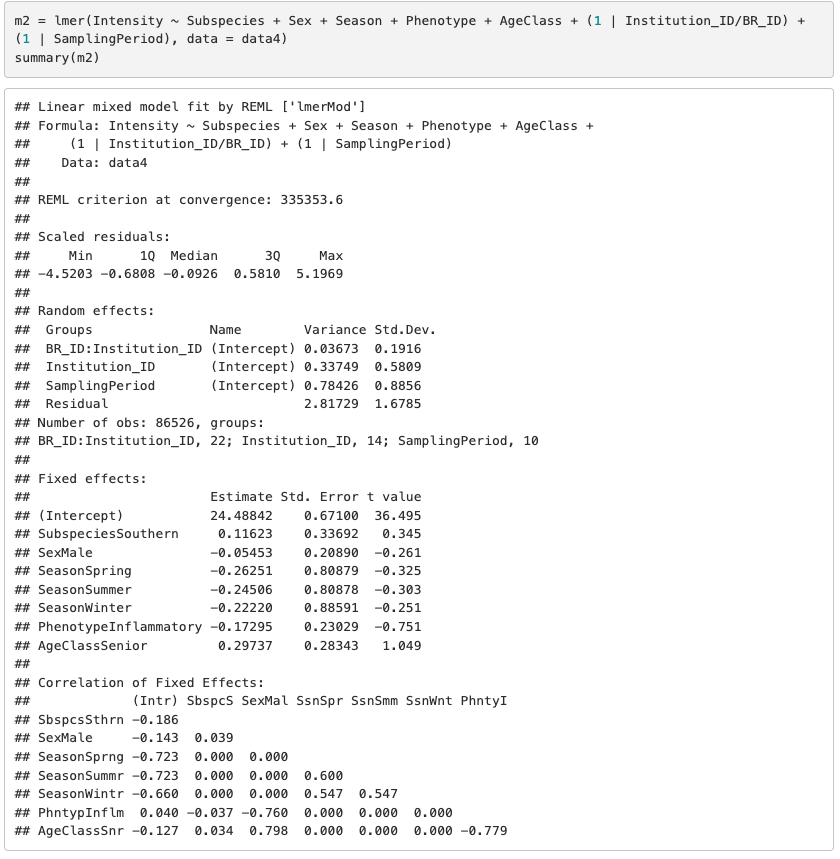


**S4B**: **Screening for potential hardwired biases within the immunoproteomic dataset.** To evaluate if any hardwired biases were present in the dataset due to unevenness in repeated measures sampling, a linear mixed effects model was fit to the data with log2(protein intensities) as the response variable, covariates subspecies, sex, season, health phenotype, and age class as fixed effects; and housing institution, sampling period, and individual animal as random effects. Graphical diagnostics generated from the model residuals indicated that the assumptions of normality and homoscedasticity were satisfactorily met (see Supplementary Data 1_CheckData.html for details), with model “m2” listed above yielding the lowest Akaike Information Criterion (AIC) value. Therefore, we proceeded with secondary bioinformatic analyses with the log2 transformed protein intensity data frame.

**Supplemental Results**

**S5A-G: PCA ordinations of immunoproteome plotted by sample and colored by metadata covariates.** Data were plotted after removing proteins with >25% missing values and imputing. We did not detect strong signatures by A) individual, B) institution, C) sex, D) subspecies, E) health status, F) age class, and G) season. Lack of clusters in the health status comparison may reflect underlying changes to health status over time and suggests that black rhino PBMC proteomes are highly dynamic, vary over time (longitudinal sampling), and/or reflect improvements following medical interventions.

**
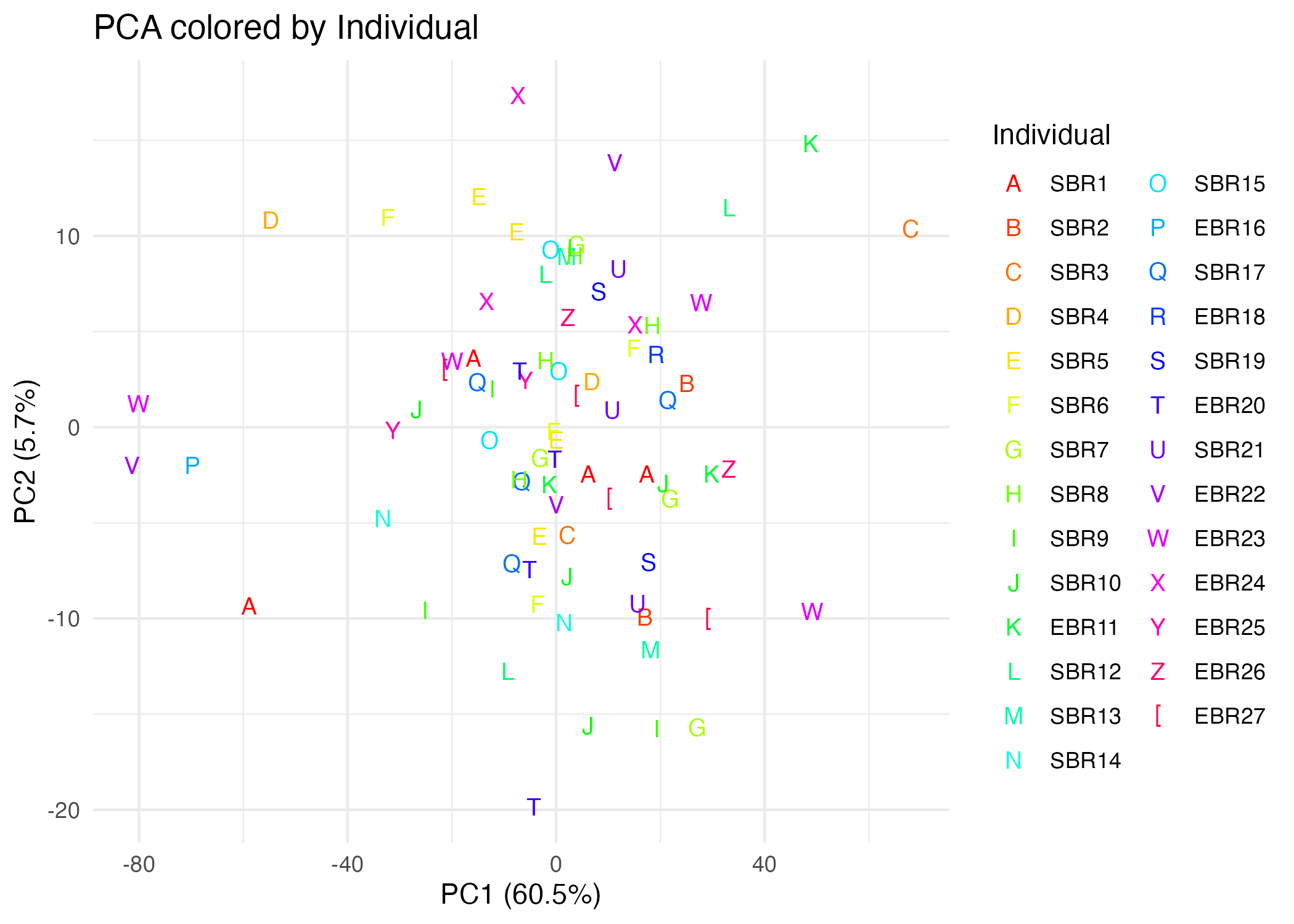
**

**S5A: PCA of PBMC proteome profiles per sample colored by individual animal.** We did not identify strong individual signatures (clustering of samples from same individuals) across longitudinal samples.

**
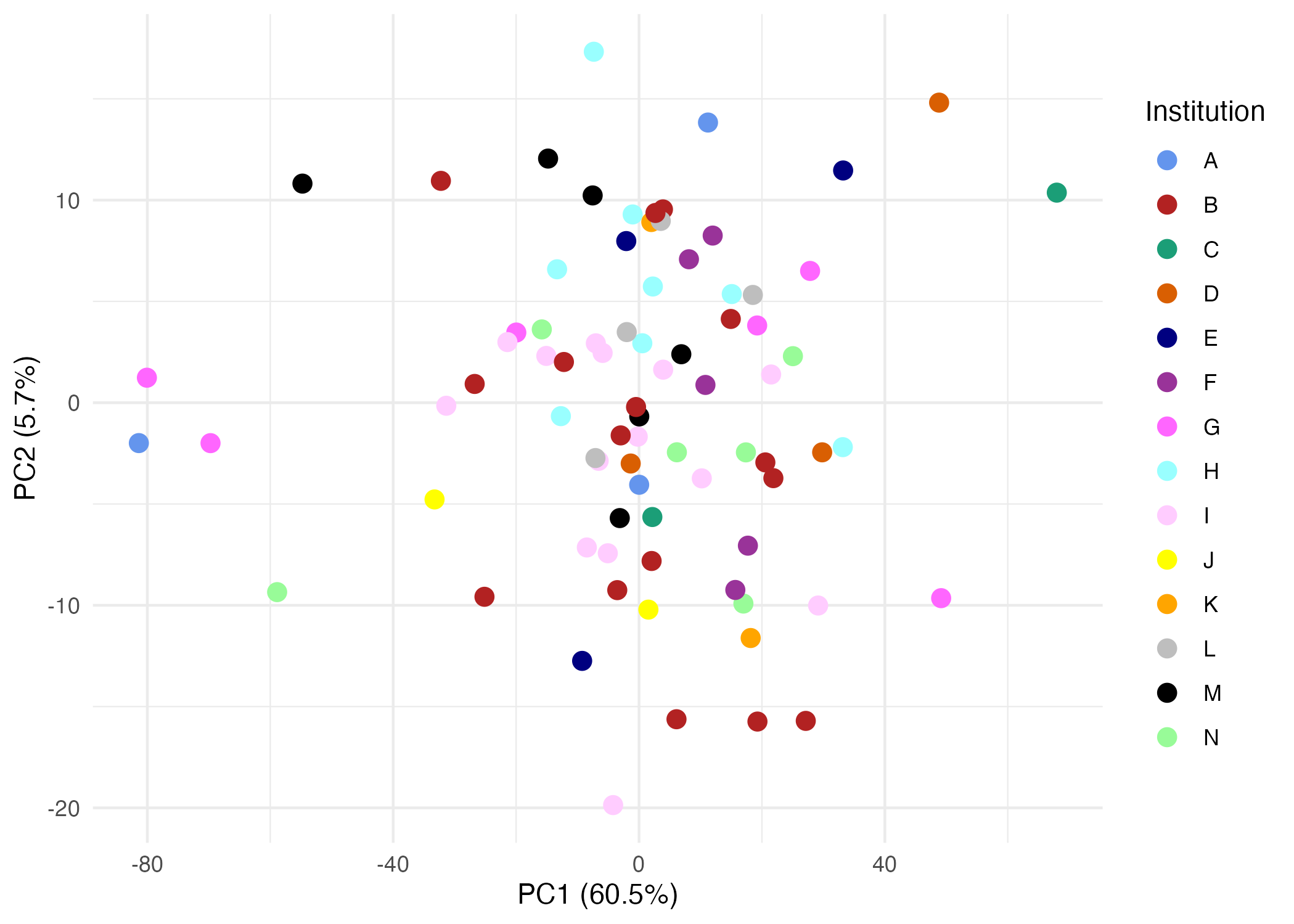
**

**S5B: PCA of PBMC proteome profiles per sample colored by housing institution.** We did not identify strong signatures of institution across samples.

**
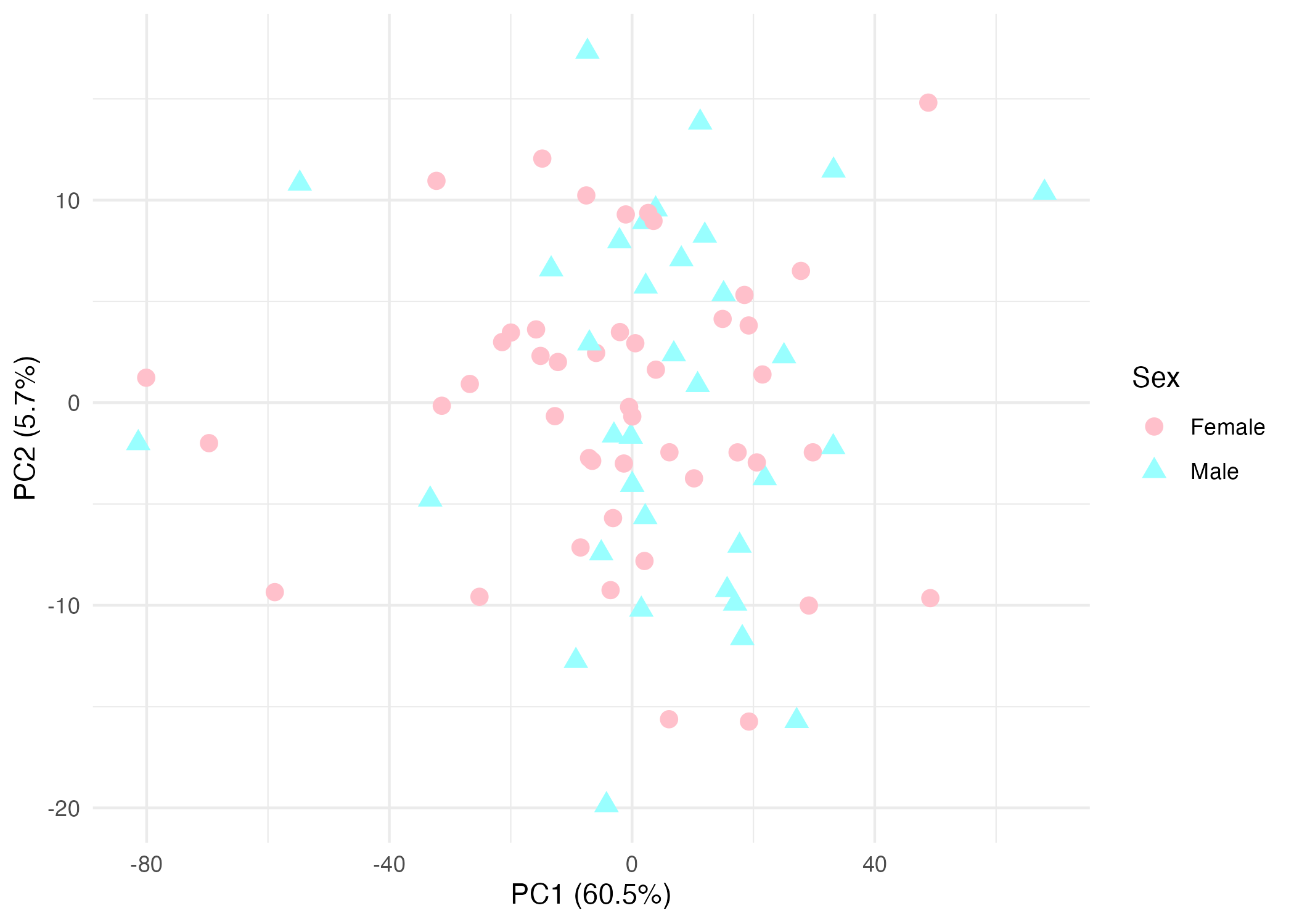
**

**S5C: PCA of PBMC proteome profiles per sample colored by sex.**

**
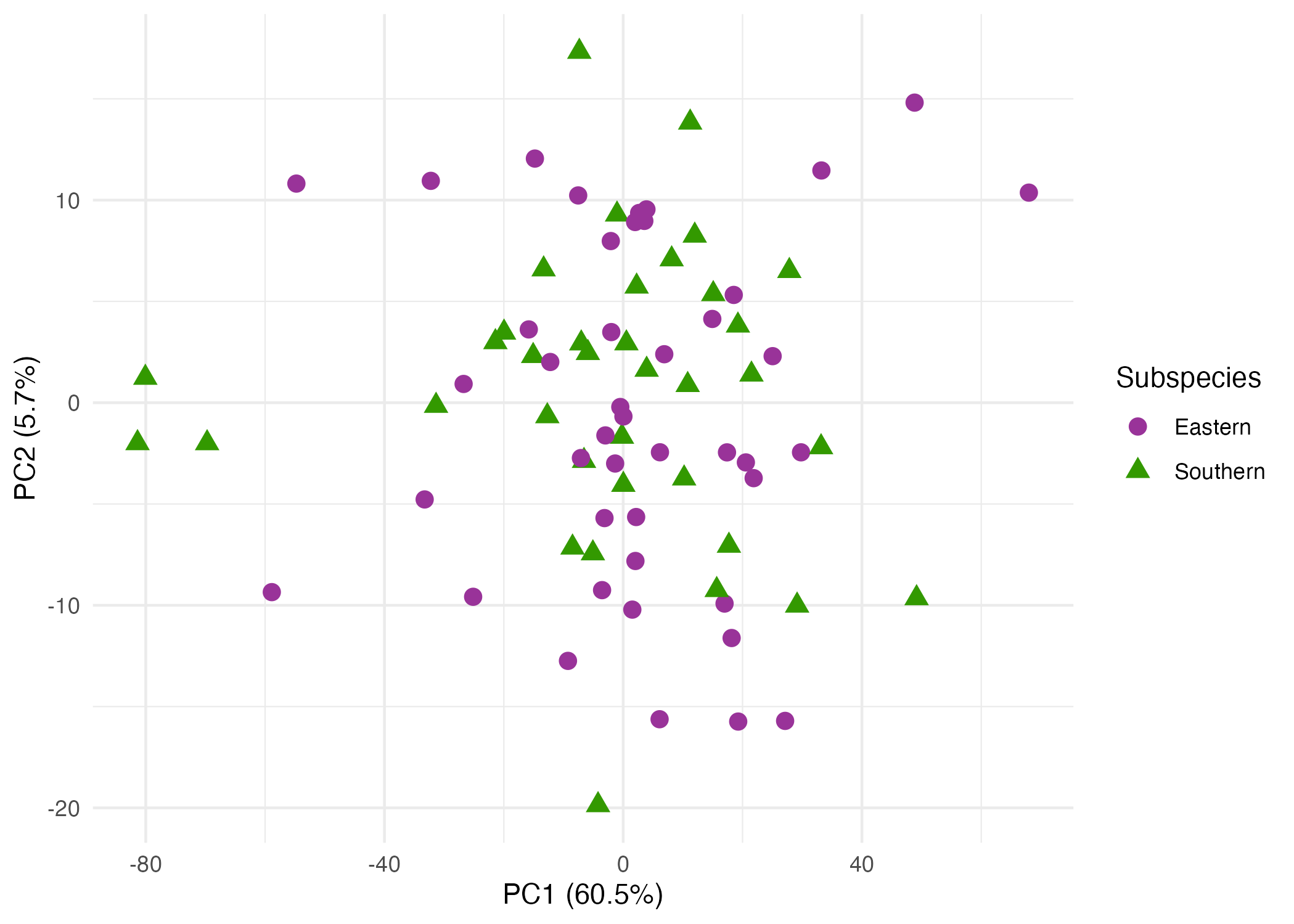
**

**S5D: PCA of PBMC proteome profiles per sample colored by subspecies.**

**
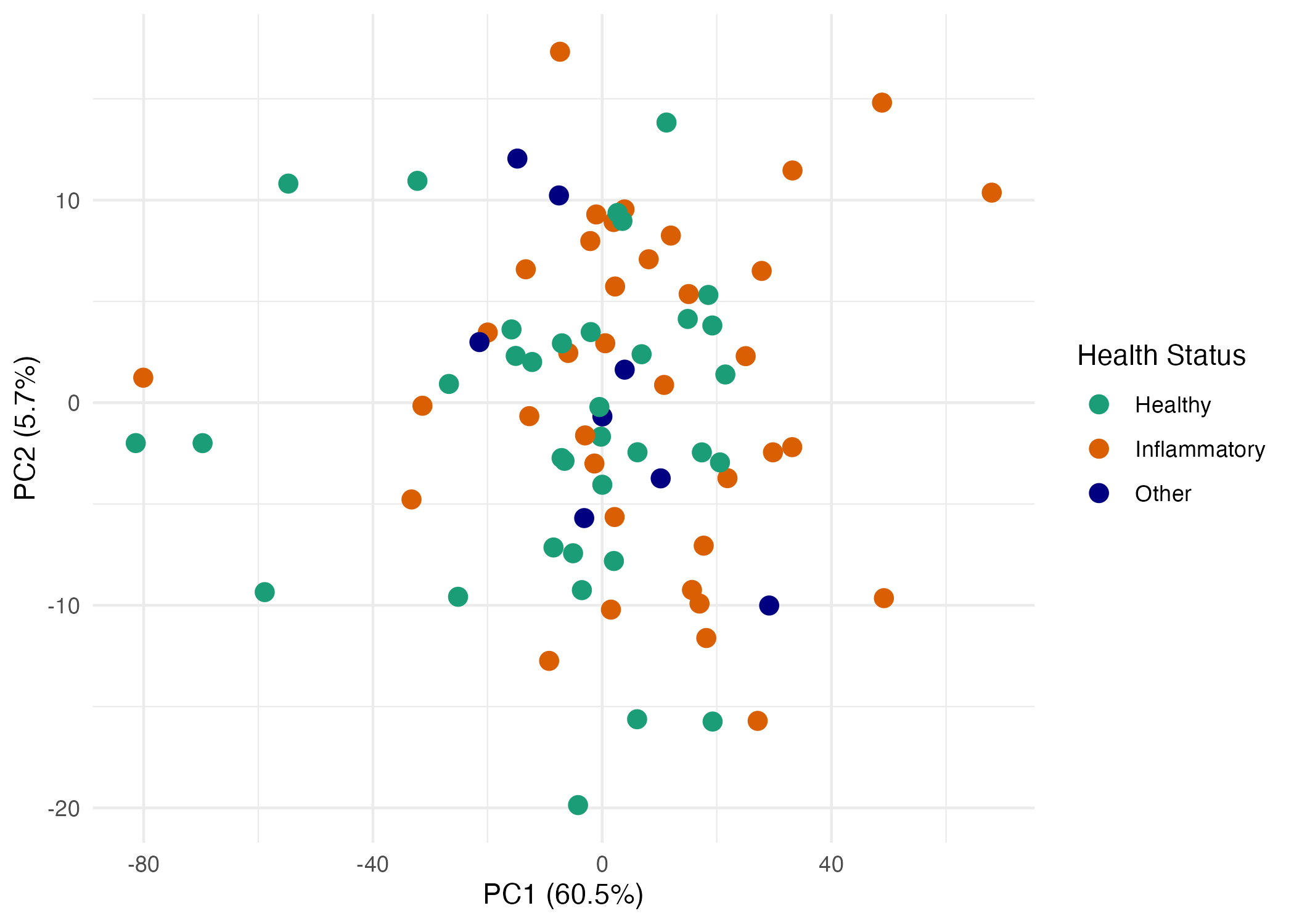
**

**S5E: PCA of PBMC proteome profiles per sample colored by clinically designated status.**

**
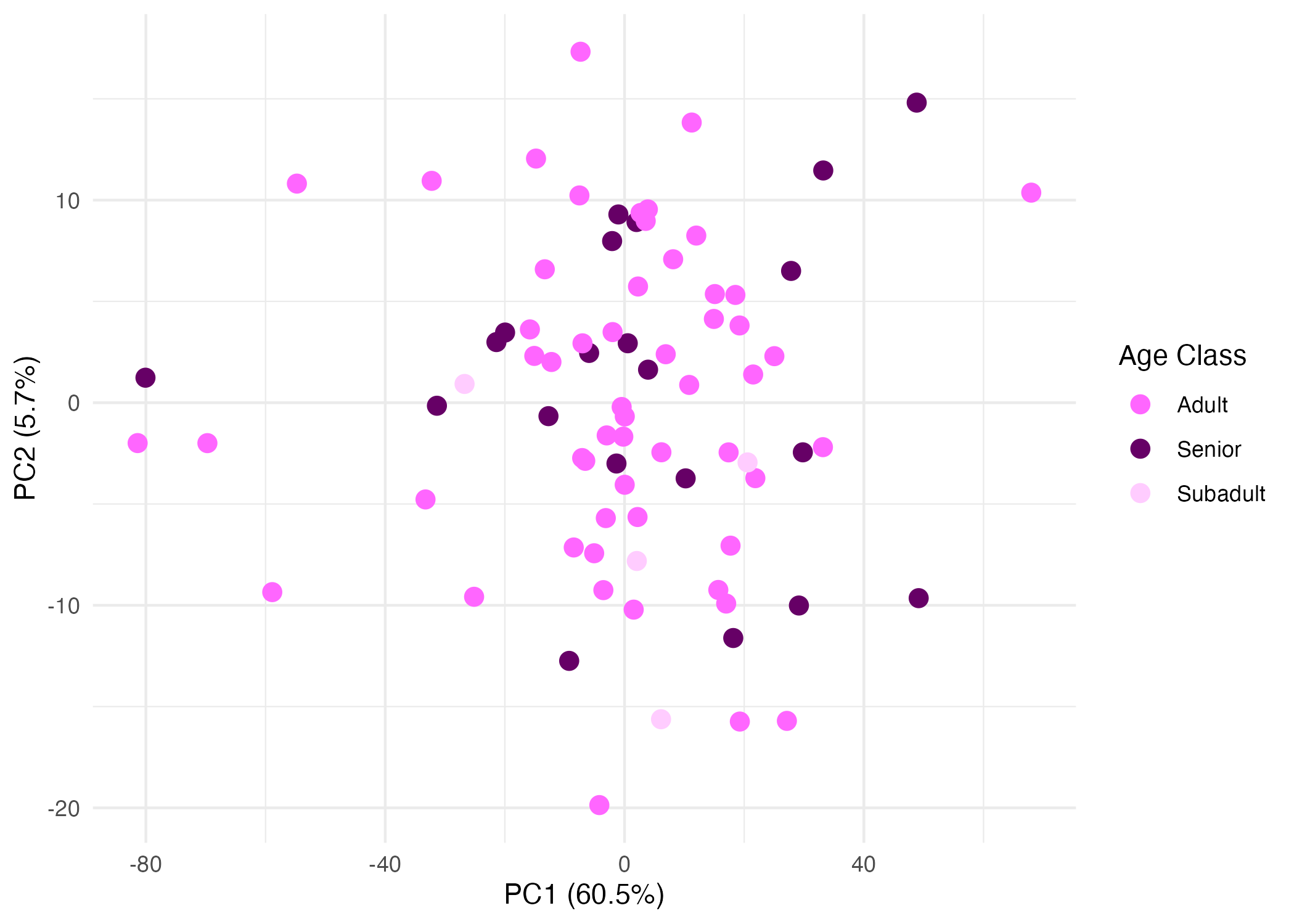
**

**S5F: PCA of PBMC proteome profiles per sample colored by age class.**

**
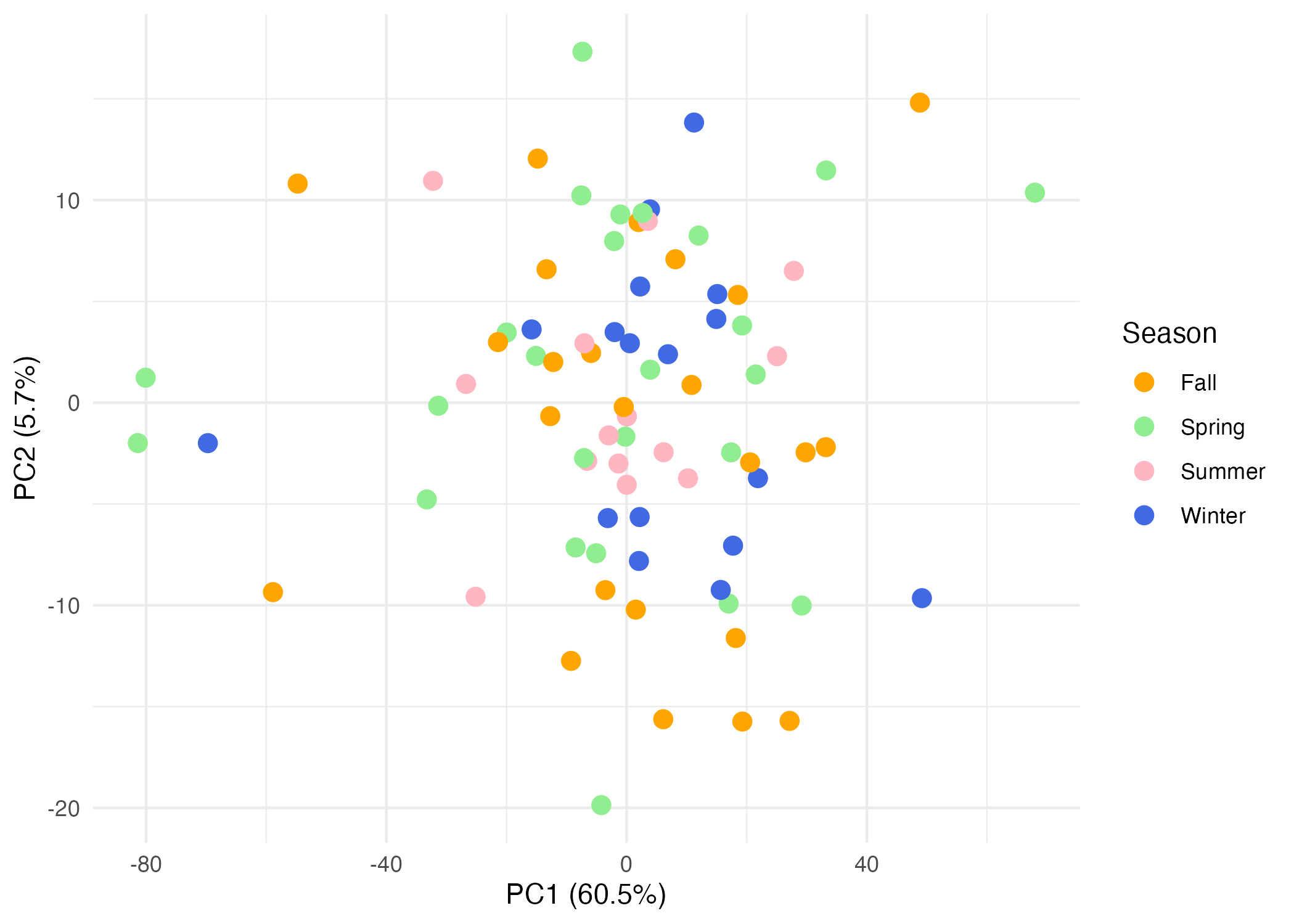
**

**S5G: PCA of PBMC proteome profiles per sample colored by season.**

**S6: Differential expression metadata driven comparisons (non-significant)**

**DiffExpressionByMetadata.xlsx** (Subspecies, Sex, Health phenotype, Age class comparisons)


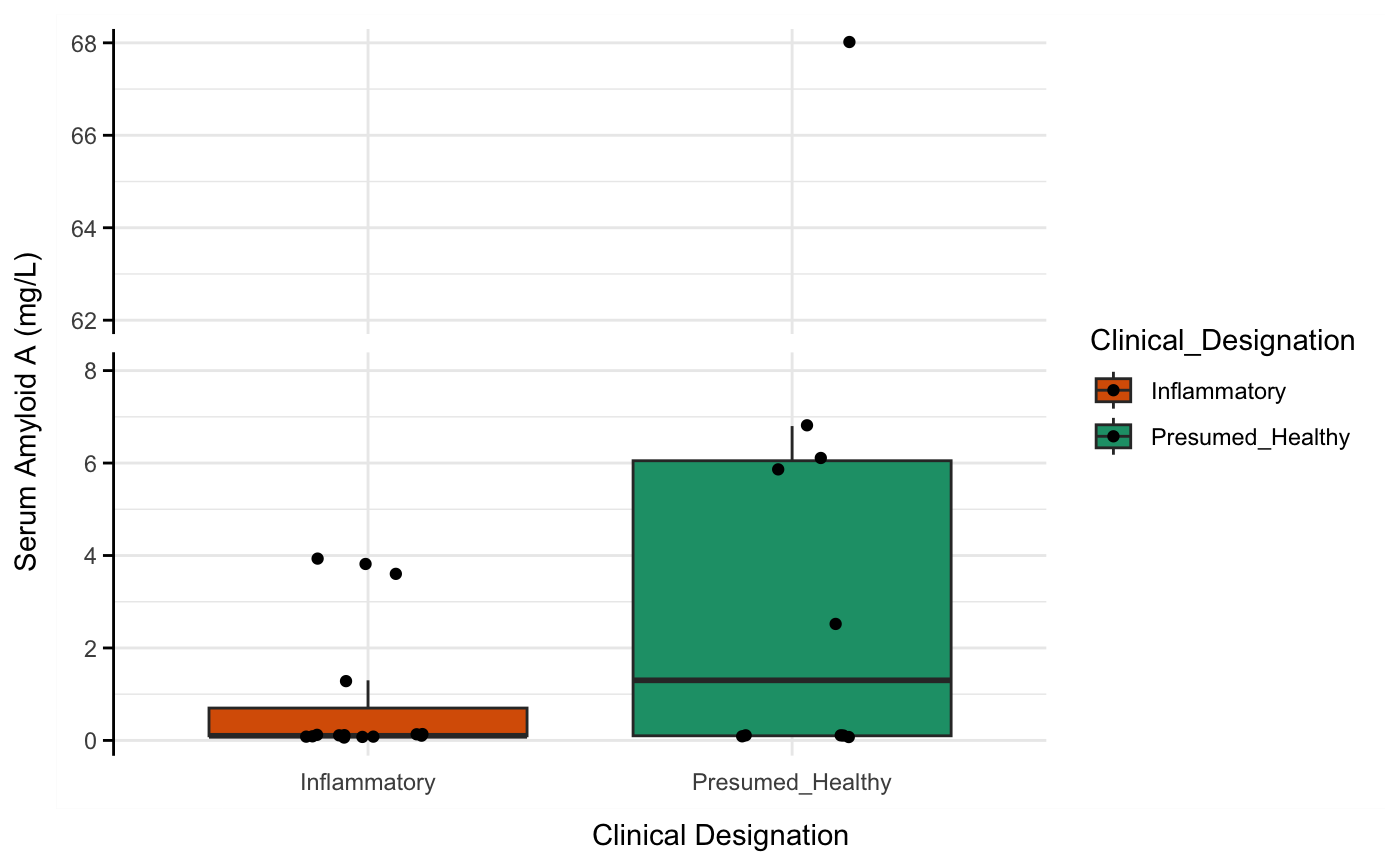


**S7: Serum amyloid A (SAA) and health status.** We attempted to validate metadata-derived clinical designations by measuring SAA an acute phase protein previously linked to pro-inflammatory states and metabolic disturbances in this species^11-14,37^. This assay was conducted at a commercial laboratory widely used by the zoo/wildlife medicine industry. Here, y-axis represents SAA (mg/L) values and x-axis represents metadata derived clinical designation (inflammatory vs presumed healthy). SAA values ranged from 0.1 to 68 (mg/L). Data was not normally distributed (Shapiro-Wilk test, W = 0.31, p <0.001). No significant differences were detected in SAA values based on clinical designations via Wilcoxon rank-sum test with continuity correction (W=50.5, P value = 0.1213). Highest SAA values detected were in presumed healthy animals, and multiple individuals in both cohorts fell within the subclinical disease SAA range previously published for clinically healthy (<1 mg/L), subclinical (1-7 mg/L), and clinically abnormal (>7 mg/L)^13^.

**S8A-E: PCA ordinations by unsupervised clustering methods.** Methods included A) K-means, B) hierarchical clustering, and C) consensus clustering. We evaluated K-means, hierarchical, and consensus clustering algorithms before choosing consensus clustering as the more robust unsupervised clustering algorithm. While k-means clustering works well for large datasets and is easy to interpret, determining the number of clusters must be done *a priori* and somewhat subjective. Hierarchical clustering does not need a fixed number of clusters to start and can measure similarity and linkage. Both k-means clustering and hierarchical clustering are sensitive to noise and outliers. Consensus clustering was able to reproducibly and consistently find stable clusters without having to determine the number of clusters a priori.

**
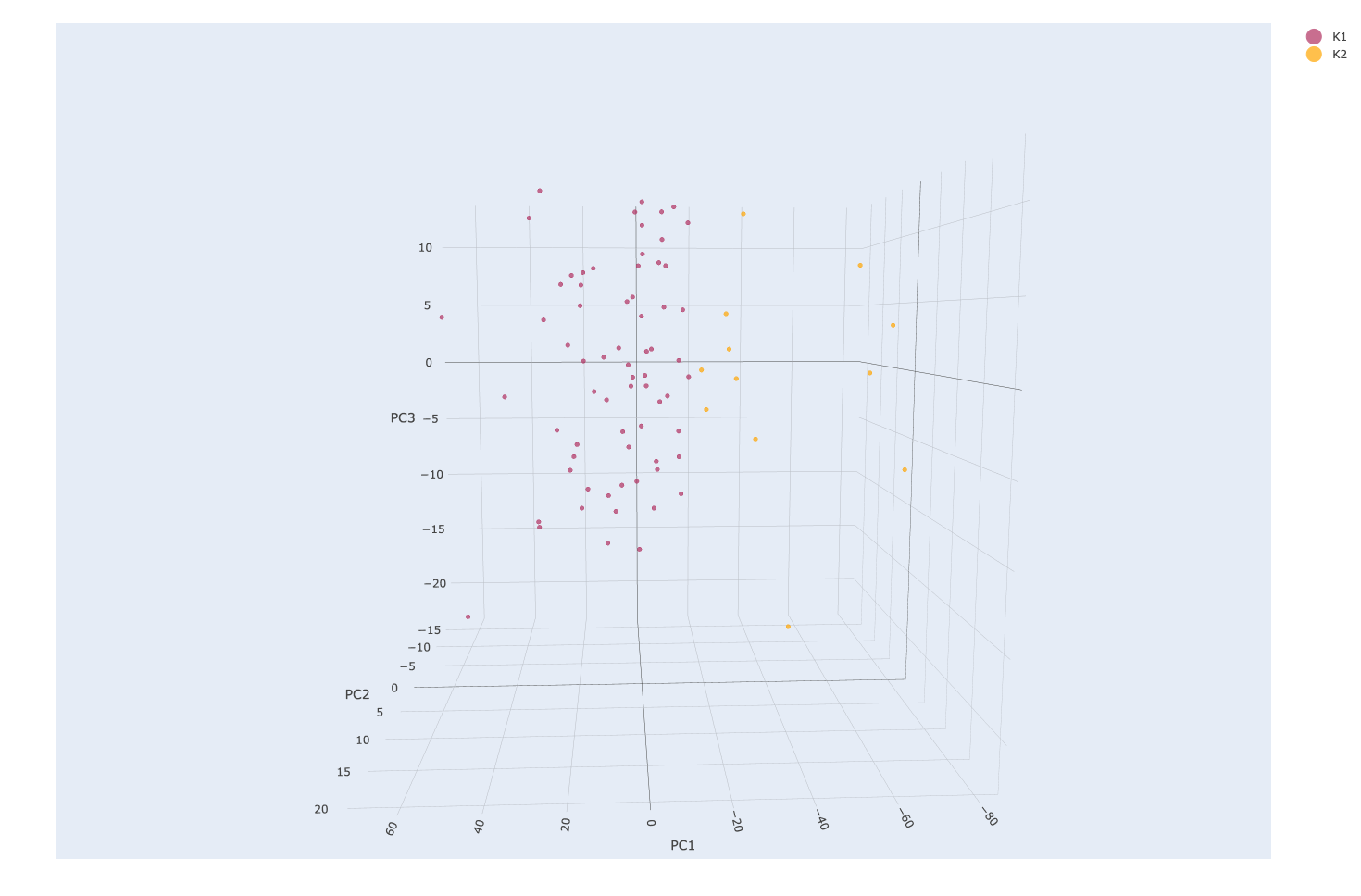
**

**S8A: PCA ordination of samples colored by K-means clustering assignment (k=2).** This clustering algorithm required the number of clusters be pre-set (k=2) and partitions the data into K non-overlapping clusters by minimizing within-cluster variance.

**
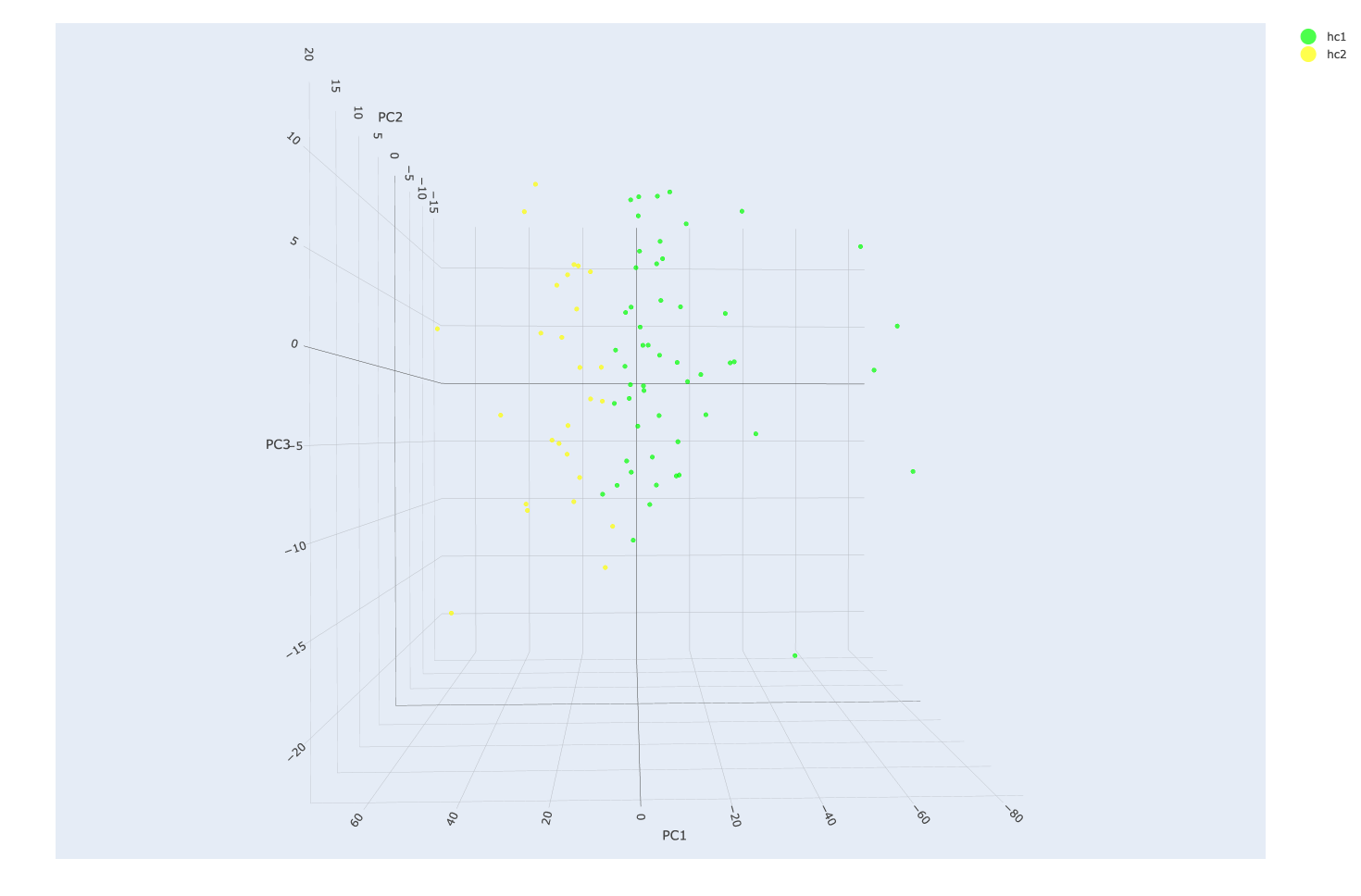
**

**S8B: PCA ordination of samples colored by hierarchical clustering assignment (k=2).** The clustering algorithm used here included Euclidean distance calculations to perform hierarchical cluster to show nested clusters using linkage criteria (without pre-specified numbers of clusters).

**
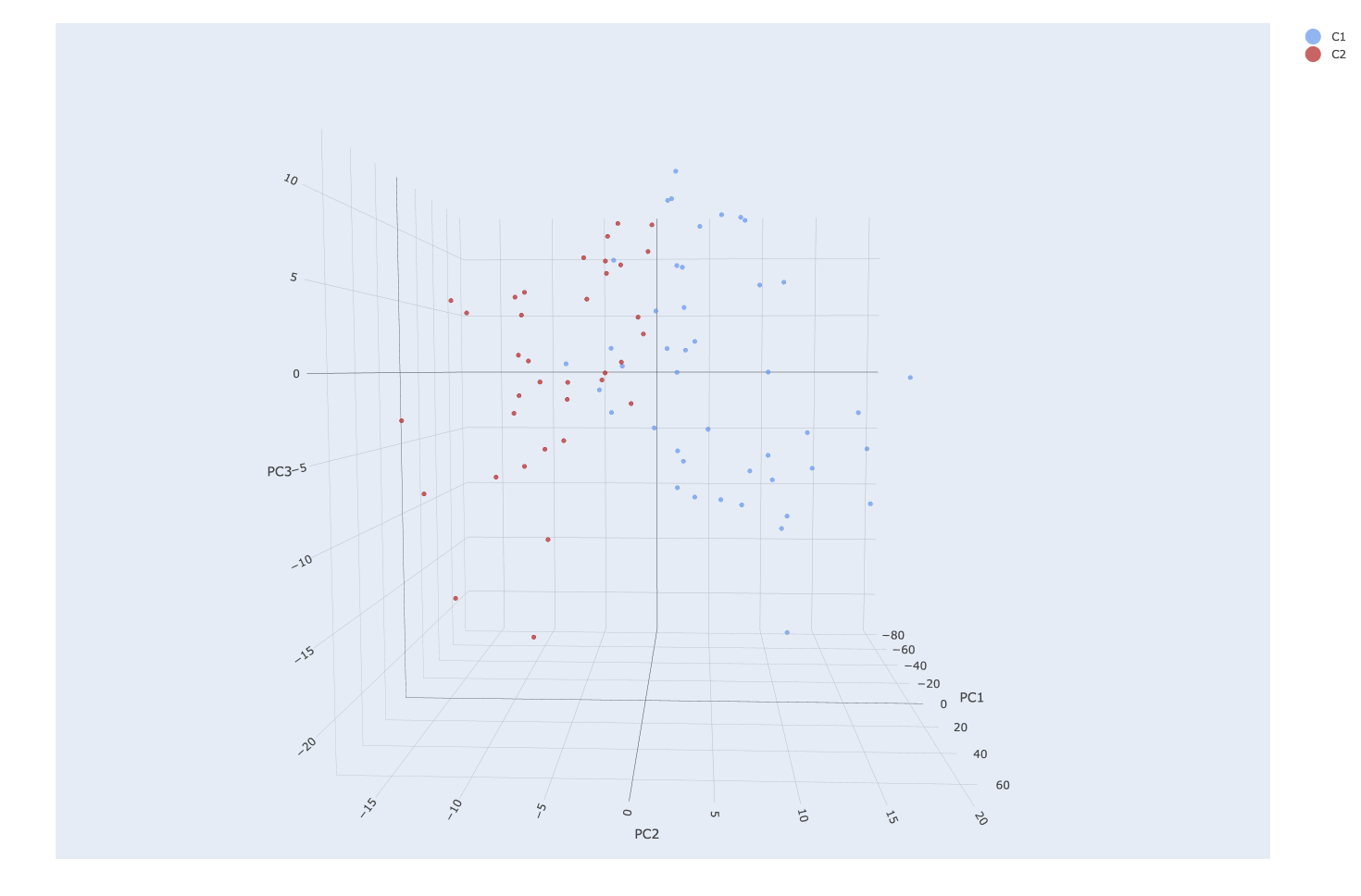
**

**S8C: PCA ordination of samples colored by consensus clustering assignment (k=2).** The clustering algorithm used here included iterative hierarchical clustering and the Consensus Cluster Plus package: ConsensusClusterPlus(dc, maxK = 4, reps = 1000, seed = 222, pItem = 0.8, pFeature=1, distance = "pearson", clusterAlg="hc"). Tnumber of clusters was not pre-specified, but rather determined after many iterations using the top 25% most variable proteins in the dataset as training data.

**
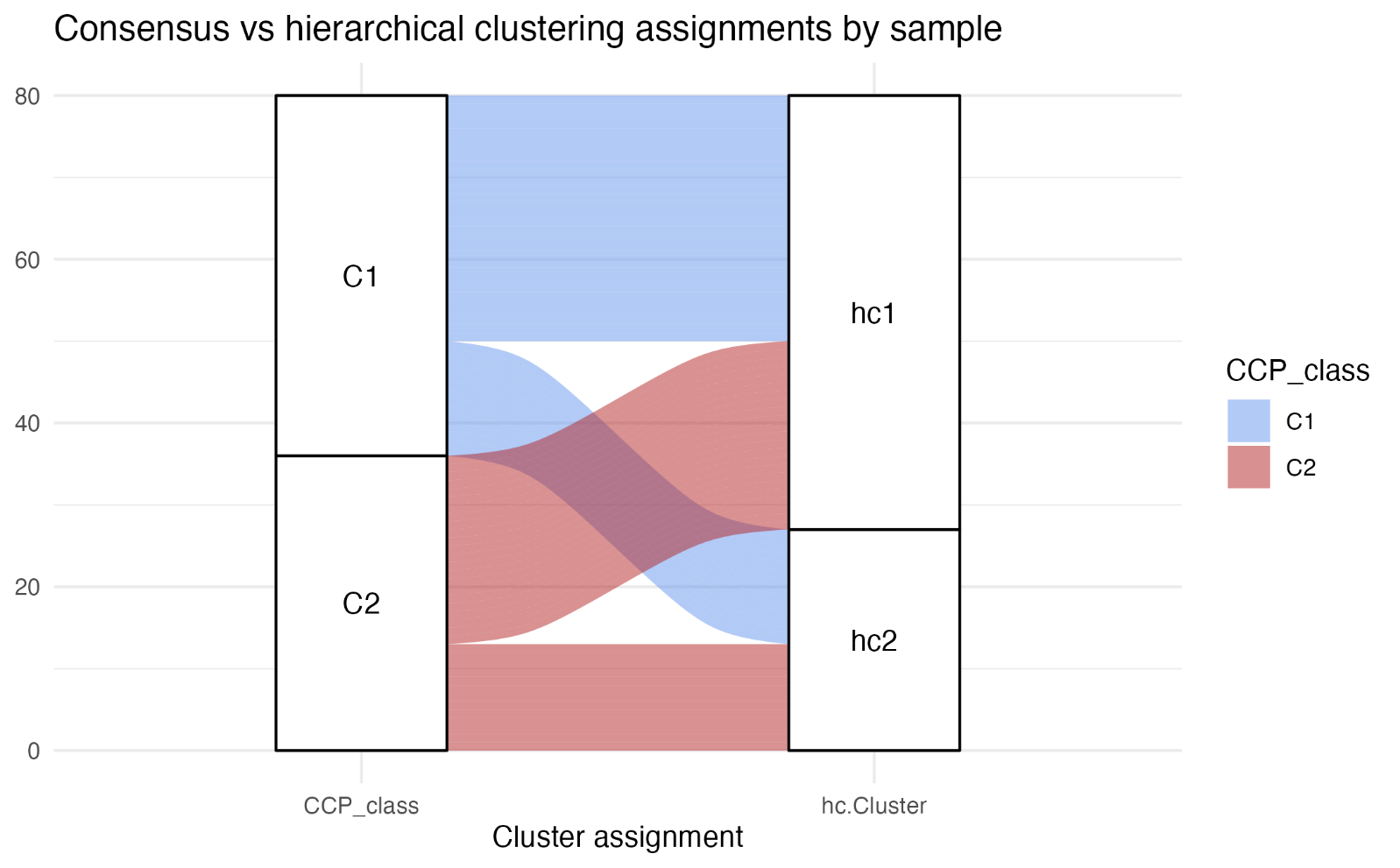
**

**S8D: Alluvial plot of consensus and hierarchical cluster assignments.** Note these distinct clustering algorithms clustered samples into two groups but cluster assignments were not identical.

**S8E: Testing for significant associations between CCP classes and metadata covariates (not significant).** Below: 2x2 contingency tables for Fisher’s exact test evaluating significant associations between Consensus Cluster Plus (CCP) class designations and metadata covariates.

| class | Subspecies | Freq |
| --- | --- | --- |
| 1 | Eastern | 26 |
| 2 | Eastern | 18 |
| 1 | Southern | 18 |
| 2 | Southern | 18 |

No significant associations between subspecies and CCP class (P value = 0.5, OR: 1.44)

| class | Sex | Freq |
| --- | --- | --- |
| 1 | Female | 26 |
| 2 | Female | 20 |
| 1 | Male | 18 |
| 2 | Male | 16 |

No significant associations between sex and CCP class (P value = 0.0.82, OR: 1.15)

| class | Phenotype | Freq |
| --- | --- | --- |
| 1 | Healthy | 21 |
| 2 | Healthy | 14 |
| 1 | Inflammatory | 17 |
| 2 | Inflammatory | 20 |

No significant associations between phenotype and CCP class (P value = 0.25, OR: 1.75)

| class | AgeClass | Freq |
| --- | --- | --- |
| 1 | Adult | 30 |
| 2 | Adult | 25 |
| 1 | Senior | 11 |
| 2 | Senior | 10 |

No significant associations between age class and CCP class (P value = 1.0, OR: 1.09)

**S9: Differential expression of proteins driving naturally emerging groups (CCP classes).** Differential expression topTable from limma in CCP class comparison. Direction of expression based on class 2, where direction of expression for CCP class logFC (C2/C1).

**S10A: Evidence for temporal fluctuations in inflammatory phenotypes.** Class switching was detected in 22 out of 27 animals.

| BR_ID | Quarter | class |
| --- | --- | --- |
| EBR01 | 1 | C1 |
| EBR01 | 2 | C2 |
| EBR01 | 3 | C1 |
| EBR02 | 1 | C2 |
| EBR02 | 2 | C2 |
| EBR02 | 3 | C1 |
| EBR03 | 1 | C2 |
| EBR03 | 2 | C1 |
| EBR04 | 1 | C1 |
| EBR04 | 2 | C1 |
| EBR05 | 1 | C1 |
| EBR05 | 2 | C1 |
| EBR05 | 3 | C1 |
| EBR05 | 4 | C2 |
| EBR06 | 1 | C1 |
| EBR06 | 2 | C2 |
| EBR07 | 1 | C1 |
| EBR07 | 2 | C2 |
| EBR08 | 1 | C2 |
| EBR08 | 2 | C1 |
| EBR09 | 1 | C1 |
| EBR09 | 2 | C2 |
| EBR09 | 3 | C1 |
| EBR09 | 4 | C2 |
| EBR10 | 1 | C2 |
| EBR10 | 2 | C2 |
| EBR10 | 3 | C2 |
| EBR10 | 4 | C1 |
| EBR11 | 1 | C2 |
| EBR11 | 2 | C1 |
| EBR11 | 3 | C1 |
| EBR11 | 4 | C1 |
| EBR12 | 1 | C1 |
| EBR12 | 2 | C2 |
| EBR12 | 3 | C2 |
| EBR12 | 4 | C2 |
| EBR13 | 1 | C2 |
| EBR13 | 2 | C2 |
| EBR13 | 3 | C1 |
| EBR13 | 4 | C1 |
| EBR14 | 1 | C1 |
| EBR14 | 2 | C1 |
| EBR14 | 3 | C2 |
| EBR14 | 4 | C1 |
| SBR01 | 1 | C1 |
| SBR01 | 2 | C2 |
| SBR01 | 3 | C2 |
| SBR02 | 1 | C2 |
| SBR02 | 2 | C1 |
| SBR02 | 3 | C2 |
| SBR02 | 4 | C2 |
| SBR03 | 1 | C1 |
| SBR03 | 2 | C2 |
| SBR04 | 1 | C1 |
| SBR04 | 2 | C2 |
| SBR05 | 1 | C1 |
| SBR05 | 2 | C1 |
| SBR05 | 3 | C1 |
| SBR05 | 4 | C1 |
| SBR06 | 1 | C1 |
| SBR07 | 1 | C2 |
| SBR07 | 2 | C1 |
| SBR07 | 3 | C2 |
| SBR07 | 4 | C1 |
| SBR08 | 1 | C2 |
| SBR09 | 1 | C2 |
| SBR09 | 2 | C1 |
| SBR10 | 1 | C1 |
| SBR10 | 2 | C2 |
| SBR10 | 3 | C1 |
| SBR10 | 4 | C1 |
| SBR11 | 1 | C2 |
| SBR11 | 2 | C2 |
| SBR11 | 3 | C1 |
| SBR12 | 1 | C1 |
| SBR12 | 2 | C2 |
| SBR12 | 3 | C1 |
| SBR13 | 1 | C2 |
| SBR13 | 2 | C2 |
| SBR13 | 3 | C2 |


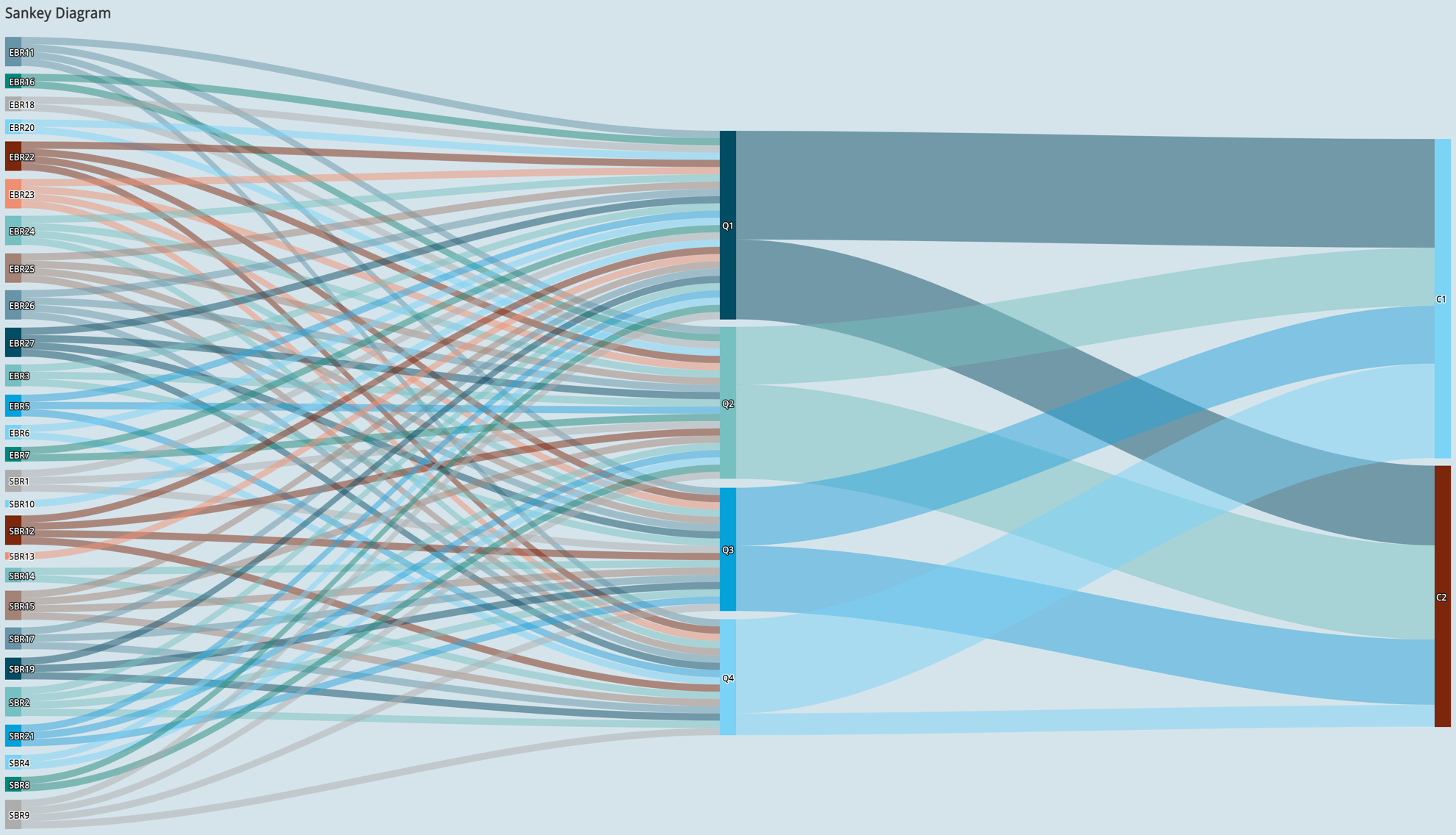


**S10B: Sankey diagram of CCP class switching.** Left: BR_ID = black rhino identifier where EBR = eastern and SBR = southern black rhino, center: longitudinal sampling event (Q1-4), and right: CCP class assignment.


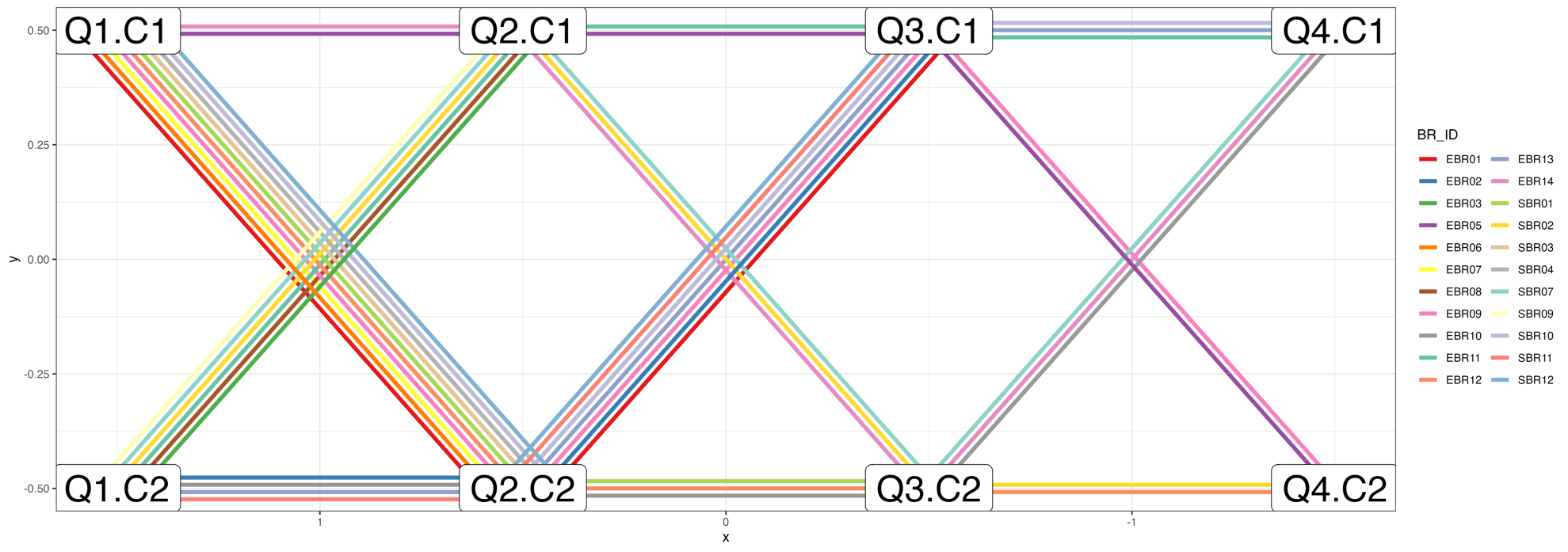


**S10C: Network diagram of CCP class switching**. PBMC samples are plotted by longitudinal sampling event and CCP class assignment. BR_ID represents black rhino identifier where EBR = eastern and SBR = southern black rhino.

**S11A: Functional profile data for Figure 3 reflecting biological interpretation of naturally emerging groups (CCP classes).**

**tpClassRE_DEG_Combo.tsv**

**S11B: Evidence for LPS as an annotation redundancy, rather than an LPS-driven immune response.** After identifying 43 proteins defining CCP classes (manuscript Figure 2 & Supplementary Information S9), we performed functional profiling (manuscript Figure 3) where neutrophil degranulation emerged as the most enriched pathway (gene ratio: 29/43, adjusted P value = 1.49 x 10^-35^), where genes detected encode for experimentally detected proteins. With other notable pathways including GOCC vesicle lumen (gene ratio: 328/19525, adjusted P value = 1.34 x 10^-30^), and GSE4748 LPS related pathway (gene ratio: 14/43, adjusted P value = 5.06 x 10^-16^) also detected. Above, we provide clarifying evidence that 12 of the 14 genes detected in the GSE4748 LPS related pathway are identical to a subset of the 29 genes detected in the neutrophil degranulation pathway. The 2 of 14 genes not identical to the subset of genes from the neutrophil degranulation pathway gene list included NCF4 and AZU1. Expression of NCF4 and AZU1 is not LPS-specific and can be induced by many inflammatory or microbial signals. Taken together with the experimental findings that showed no difference in circulating LPS levels by CCP classes (manuscript Figure 4, Supplementary Information S13), this suggests that the LPS related pathway likely reflects an annotation redundancy and is better interpreted as a general signature of neutrophil activation rather than evidence of LPS-mediated immune stimulus.

**S12: Heatmaps**: The following figures utilize gene identifiers that encode for experimentally detected proteins.

**S12A: Heatmap of proteins driving naturally emerging groups (CCP classes).**

**S12B: Heatmap of proteins upregulated in CCP class 2 with known roles in periodontitis.**

**S12C: Heatmap of proteins upregulated in CCP class 2 with known roles in gastrointestinal dysbiosis.**

**S12D: Heatmap of proteins upregulated in CCP class 2 with known roles in systemic inflammation.**

**S12E: Literature Cited list for proteins with known roles in reported disease phenotypes.**


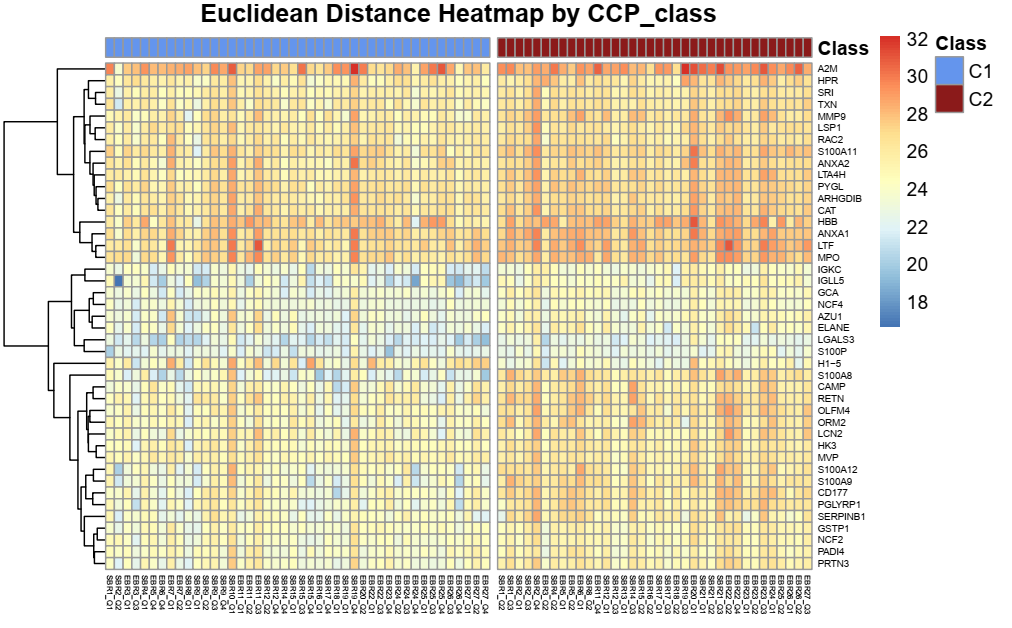


**S12A: Heatmap of differentially expressed proteins in CCP class 1 and 2 comparison.** Euclidean distance heatmap used a hierarchical clustering-based dendrogram of all differentially expressed proteins between CCP class 1 and 2. Dendrogram results indicate AM2 (adrenomedullin 2) clustered separately from all other proteins throughout all samples (n=80).

**
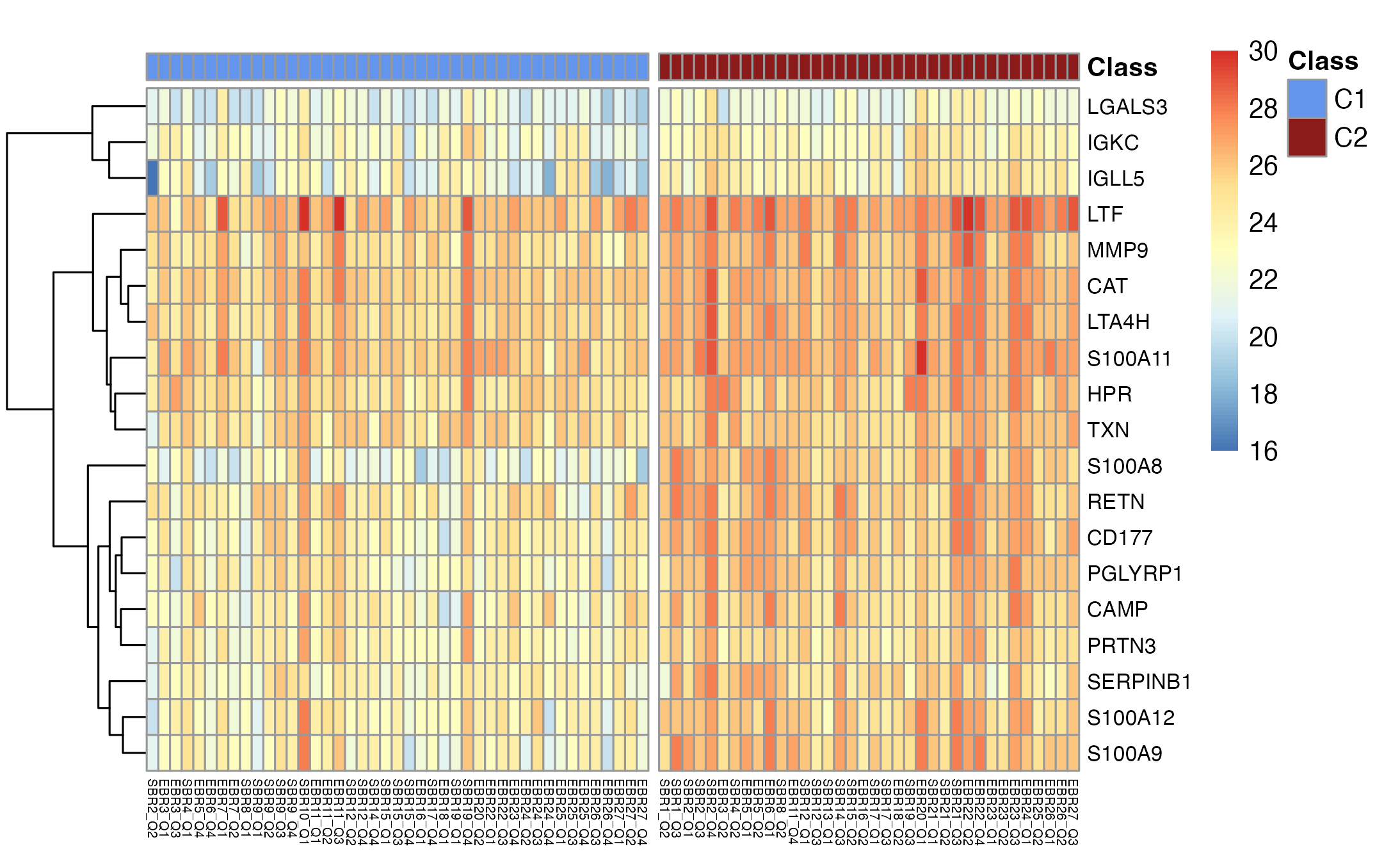
**

**S12B: Heatmap of proteins upregulated in CCP class 2 with known roles in periodontitis.**

Euclidean distance heatmap used a hierarchical clustering-based dendrogram to show presence of proteins upregulated in periodontitis. Proteins with known roles in periodontitis include: S100A8 and S100A9^1^, CD177^2^, PGLYRP1^3^ ^4^, S100A12^5^, IGLL5^6^, MMP9^7^, LTA4H^8^, SERPINB1^9^, CAMP^10^, PRTN3^11^, HPR^12^, S100A11^13^, RETN^14^, LGALS3^15^, IGKC^16^, CAT^17^, LTF^18^, and TXN^19^.


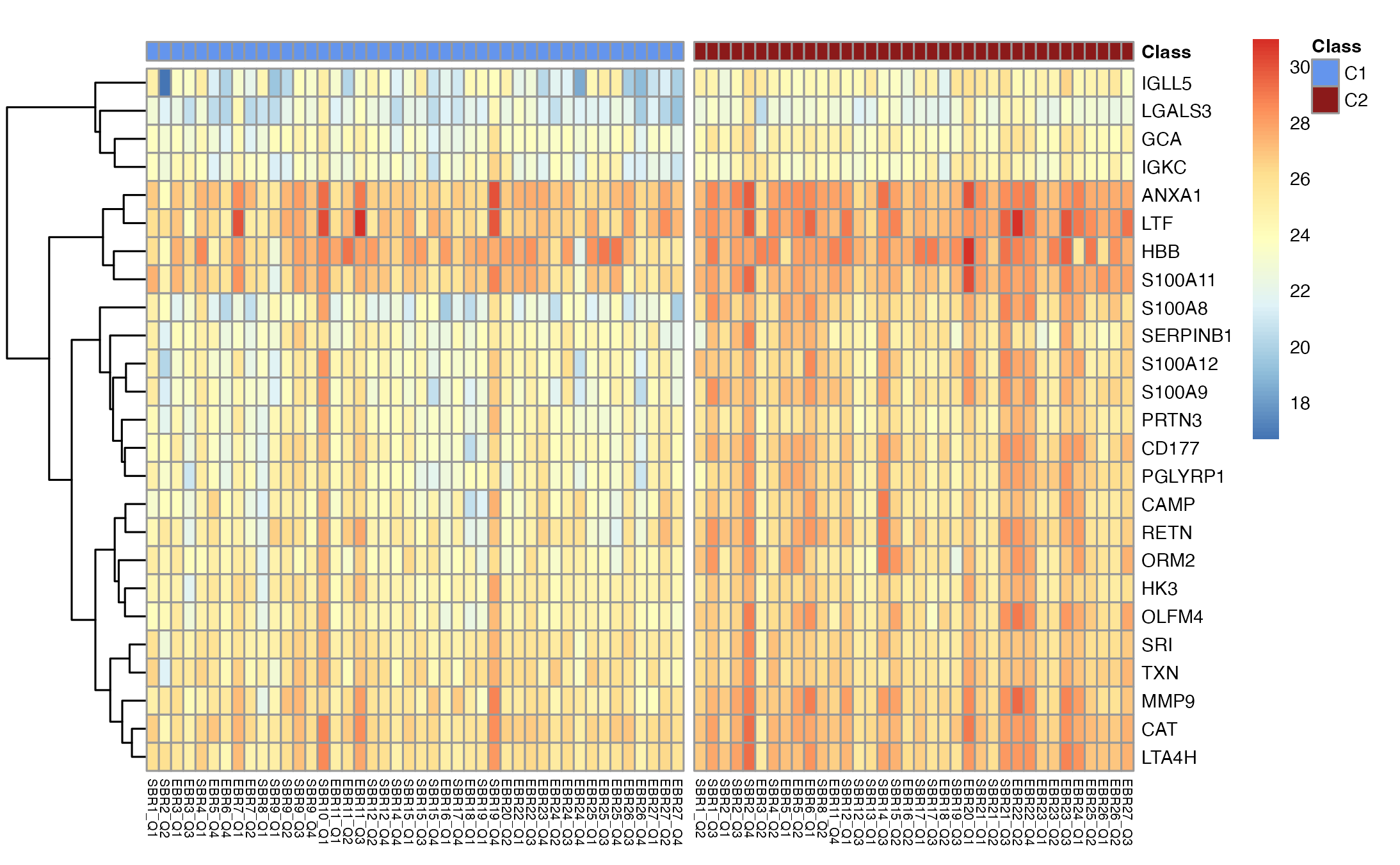


**S12C: Heatmap of proteins upregulated in CCP class 2 with known roles in gastrointestinal dysbiosis.** Euclidean distance heatmap used a hierarchical clustering-based dendrogram to show presence of proteins upregulated in gastrointestinal dysbiosis. Proteins with known roles in gastrointestinal dysbiosis include: S100A8^20^, S100A9^21^, CD177 and OLFM4^22^, PGLYRP1^23^, S100A12^24^, IGLL5^25^, MMP9^26^, LTA4H^27^, SERPINB1^28^, ORM2^29^, GCA^30^, CAMP^31^, PRTN3^32^, S100A11^33^, RETN^34^, HBB^35^, LGALS3^36^, IGKC^16^, HK3^37^, A2M^38^, ANXA1^39^, CAT^40^, SRI^41^, LTF^42^ and TXN^43^.


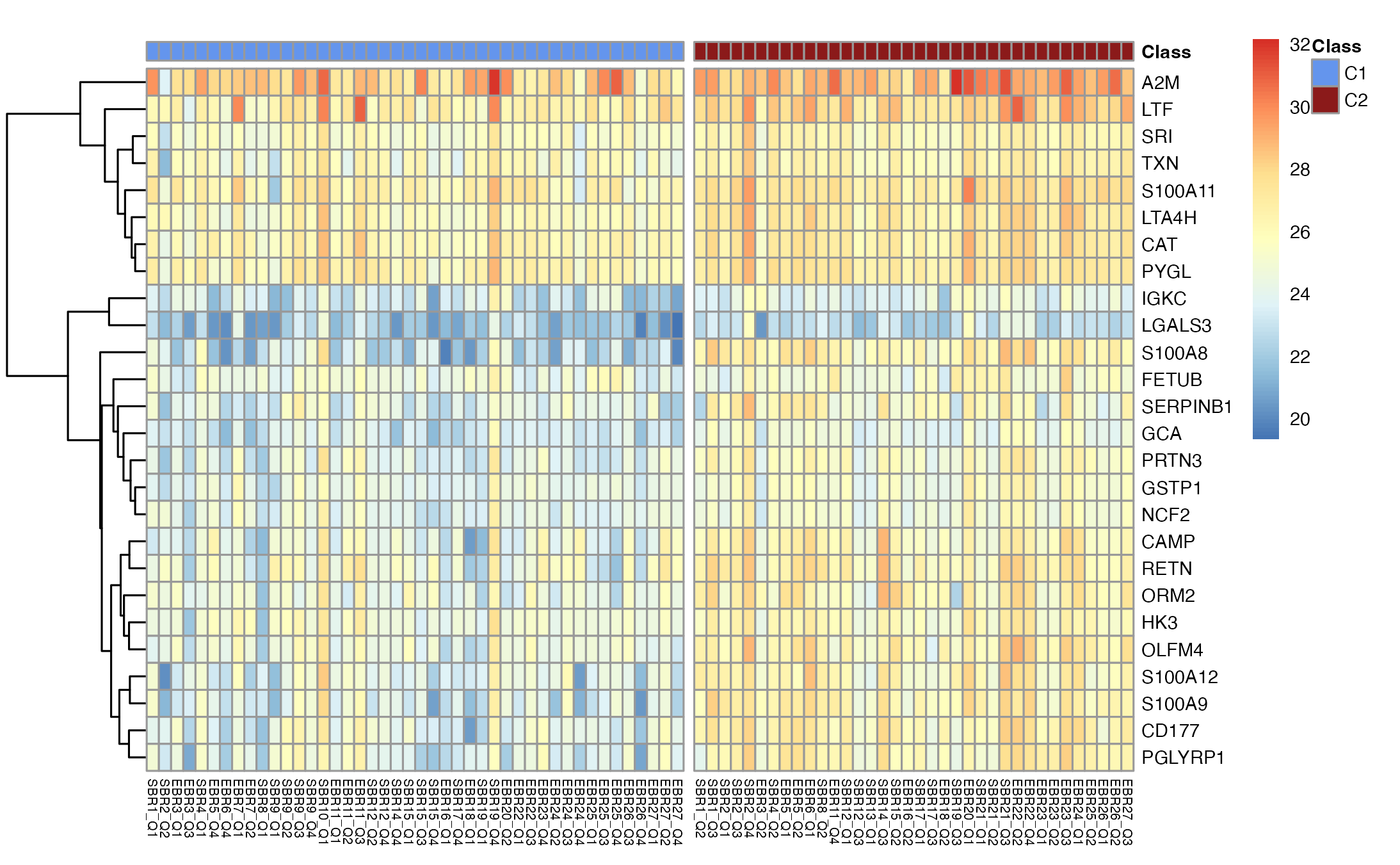


**S12D: Heatmap of proteins upregulated in CCP class 2 with known roles in systemic inflammation.** Euclidean distance heatmap used a hierarchical clustering-based dendrogram to show presence of proteins upregulated in systemic inflammation. Proteins with known roles in systemic inflammation include: S100A8^44^, S100A9^45^, CD177^46^, PGLYRP1^47^, S100A12^48^, OLFM4^49^, LTA4H^50^, SERPINB1^51^, ORM2^52^, GCA^30^, CAMP^53^, PRTN3^54^, S100A11^55^, GSTP1^56^, RETN^57^, LGALS3^58^, IGKC^59^, HK3^60^, A2M^61^, ANXA1^62^, CAT^63^, SRI^64^, LTF^42^, TXN^65^, NCF2^66^, and PYGL^67^.

**S12E: Literature Cited list for proteins with known roles in reported disease phenotypes reported in S8 figures.**

1. Maekawa, S. *et al.* RNA sequencing for ligature induced periodontitis in mice revealed important role of S100A8 and S100A9 for periodontal destruction. *Scientific Reports* **9**, 14663 (2019).

2. Dahlstrand Rudin, A. *et al.* The neutrophil subset defined by CD177 expression is preferentially recruited to gingival crevicular fluid in periodontitis. *Journal of Leukocyte Biology* **109**, 349–362 (2021).

3. Silbereisen, A. *et al.* Regulation of PGLYRP1 and TREM-1 during progression and resolution of gingival inflammation. *JDR Clinical & Translational Research* **4**, 352–359 (2019).

4. Yucel, Z. P. K. *et al.* Salivary biomarkers in the context of gingival inflammation in children with cystic fibrosis. *Journal of Periodontology* **91**, 1339–1347 (2020).

5. Lira-Junior, R. *et al.* S100A12 expression is modulated during monocyte differentiation and reflects periodontitis severity. *Frontiers in Immunology* **11**, (2020).

6. Lundmark, A. *et al.* Gene expression profiling of periodontitis-affected gingival tissue by spatial transcriptomics. *Scientific Reports* **8**, 9370 (2018).

7. Franco, C., Patricia, H.-R., Timo, S., Claudia, B. & Marcela, H. Matrix metalloproteinases as regulators of periodontal inflammation. *International Journal of Molecular Sciences* **18**, 440 (2017).

8. Baus-Domínguez, M. *et al.* Using genetics in periodontal disease to justify implant failure in down syndrome patients. *Journal of Clinical Medicine* **9**, 2525 (2020).

9. Cavalla, F. *et al.* Proteomic profiling and differential messenger RNA expression correlate HSP27 and serpin family B member 1 to apical periodontitis outcomes. *Journal of Endodontics* **43**, 1486–1493 (2017).

10. Bose, A., Narayan, S. J. & Santosh, H. N. A randomized controlled crossover trial for reinforcement of epithelial barrier function by vitamin D induction of the antimicrobial peptide cathelicidin - A novel therapeutic approach in chronic periodontitis. *RGUHS Journal of Dental Sciences* **14**, (2022).

11. Türkoğlu, O., Azarsız, E., Emingil, G., Kütükçüler, N. & Atilla, G. Are proteinase 3 and cathepsin C enzymes related to pathogenesis of periodontitis? *BioMed Research International* **2014**, e420830 (2014).

12. Foratori-Junior, G. A. *et al.* Label-free quantitative proteomic analysis reveals inflammatory pattern associated with obesity and periodontitis in pregnant women. *Metabolites* **12**, 1091 (2022).

13. Li, Q. *et al.* Proteomic analysis of human periodontal ligament cells under hypoxia. *Proteome Science* **17**, 3 (2019).

14. Devanoorkar, A., Kathariya, R., Guttiganur, N., Gopalakrishnan, D. & Bagchi, P. Resistin: A potential biomarker for periodontitis influenced diabetes mellitus and diabetes induced periodontitis. *Disease Markers* **2014**, e930206 (2014).

15. Velickovic, M. *et al.* Galectin-3, Possible role in pathogenesis of periodontal diseases and potential therapeutic target. *Frontiers in Pharmacology* **12**, (2021).

16. Xiong, Z., Fang, Y., Lu, S., Sun, Q. & Huang, J. Identification and validation of signature genes and potential therapy targets of inflammatory bowel disease and periodontitis. *Journal of Inflammation Research* **16**, 4317–4330 (2023).

17. Gölz, L. *et al.* LPS from *P. gingivalis* and hypoxia increases oxidative stress in periodontal ligament fibroblasts and contributes to periodontitis. *Mediators of Inflammation* **2014**, e986264 (2014).

18. Berlutti, F., Pilloni, A., Pietropaoli, M., Polimeni, A. & Valenti, P. Lactoferrin and oral diseases: current status and perspective in periodontitis. *Ann Stomatol (Roma)* **2**, 10–18 (2012).

19. Liu, J. *et al.* Discovering genetic linkage between periodontitis and type 1 diabetes: A bioinformatics study. *Frontiers in Genetics* **14**, (2023).

20. Bao, W., Wang, L., Liu, X. & Li, M. Predicting diagnostic biomarkers associated with immune infiltration in Crohn’s disease based on machine learning and bioinformatics. *European Journal of Medical Research* **28**, 255 (2023).

21. Dheer, R. *et al.* Microbial signatures and innate immune gene expression in lamina propria phagocytes of inflammatory bowel disease patients. *Cellular and Molecular Gastroenterology and Hepatology* **9**, 387–402 (2020).

22. Nowak, J. K. *et al.* Characterisation of the circulating transcriptomic landscape in inflammatory bowel disease provides evidence for dysregulation of multiple transcription factors including NFE2, SPI1, CEBPB, and IRF2. *Journal of Crohn’s and Colitis* **16**, 1255–1268 (2022).

23. Iida, H. *et al.* Paraimmunobiotic Bifidobacteria modulate the expression patterns of peptidoglycan recognition proteins in porcine intestinal epitheliocytes and antigen presenting cells. *Cells* **8**, 891 (2019).

24. Guo, X. *et al.* Gut microbiota is a potential biomarker in inflammatory bowel disease. *Frontiers in Nutrition* **8**, (2022).

25. Zhang, X. *et al.* Widespread protein lysine acetylation in gut microbiome and its alterations in patients with Crohn’s disease. *Nature Communications* **11**, 4120 (2020).

26. Rodrigues, D. M. *et al.* Matrix metalloproteinase 9 contributes to gut microbe homeostasis in a model of infectious colitis. *BMC Microbiology* **12**, 105 (2012).

27. Rahabi, M. *et al.* Divergent roles for macrophage C-type lectin receptors, dectin-1 and mannose receptors, in the intestinal inflammatory response. *Cell Reports* **30**, 4386-4398.e5 (2020).

28. Kriaa, A. *et al.* Serine proteases at the cutting edge of IBD: Focus on gastrointestinal inflammation. *The FASEB Journal* **34**, 7270–7282 (2020).

29. Yan, P. *et al.* Integrating the serum proteomic and fecal metaproteomic to analyze the impacts of overweight/obesity on IBD: a pilot investigation. *Clinical Proteomics* **20**, 6 (2023).

30. Su, T. *et al.* Myeloid-derived grancalcin instigates obesity-induced insulin resistance and metabolic inflammation in male mice. *Nature Communications* **15**, 97 (2024).

31. Liang, W. *et al.* Intestinal cathelicidin antimicrobial peptide shapes a protective neonatal gut microbiota against pancreatic autoimmunity. *Gastroenterology* **162**, 1288-1302.e16 (2022).

32. Soussou, S. *et al.* Serine proteases and metalloproteases are highly increased in irritable bowel syndrome Tunisian patients. *Scientific Reports* **13**, 17571 (2023).

33. Melle, C. *et al.* Different expression of calgizzarin (S100A11) in normal colonic epithelium, adenoma and colorectal carcinoma. *International Journal of Oncology* **28**, 195–200 (2006).

34. Wang, C., Li, Y., Li, S., Chen, M. & Hu, Y. Proteomics combined with RNA sequencing to screen biomarkers of sepsis. *Infection and Drug Resistance* **15**, 5575–5587 (2022).

35. Chen, L. *et al.* The landscape of isoform switches in sepsis: a multicenter cohort study. *Scientific Reports* **12**, 10276 (2022).

36. Volarevic, V. *et al.* Galectin-3 regulates indoleamine-2,3-dioxygenase-dependent cross-talk between colon-infiltrating dendritic cells and T regulatory cells and may represent a valuable biomarker for monitoring the progression of ulcerative colitis. *Cells* **8**, 709 (2019).

37. Hinrichsen, F. *et al.* Microbial regulation of hexokinase 2 links mitochondrial metabolism and cell death in colitis. *Cell Metabolism* **33**, 2355-2366.e8 (2021).

38. Martínez-Herrero, S. & Martínez, A. Adrenomedullin: Not just another gastrointestinal peptide. *Biomolecules* **12**, 156 (2022).

39. Mui, L., Martin, C. M., Tschirhart, B. J. & Feng, Q. Therapeutic potential of annexins in sepsis and COVID-19. *Frontiers in Pharmacology* **12**, (2021).

40. Wang, W. *et al.* Dietary catalase supplementation alleviates deoxynivalenol-induced oxidative stress and gut microbiota dysbiosis in broiler chickens. *Toxins* **14**, 830 (2022).

41. Deng, L. *et al.* Upregulation of soluble resistance-related calcium-binding protein (sorcin) in gastric cancer. *Medical Oncology* **27**, 1102–1108 (2010).

42. Kruzel, M. L., Zimecki, M. & Actor, J. K. Lactoferrin in a context of inflammation-induced pathology. *Frontiers in Immunology* **8**, (2017).

43. Zhu, M., Dagah, O. M. A., Silaa, B. B. & Lu, J. Thioredoxin/glutaredoxin systems and gut microbiota in NAFLD: Interplay, mechanism, and therapeutical potential. *Antioxidants* **12**, 1680 (2023).

44. Ryckman, C., Vandal, K., Rouleau, P., Talbot, M. & Tessier, P. A. Proinflammatory activities of S100: Proteins S100A8, S100A9, and S100A8/A9 induce neutrophil chemotaxis and adhesion 1. *The Journal of Immunology* **170**, 3233–3242 (2003).

45. Henke, M. O. *et al.* Up-regulation of S100a8 and S100a9 protein in bronchial epithelial cells by lipopolysaccharide. *Experimental Lung Research* **32**, 331–347 (2006).

46. Saha, R. *et al.* Inflammatory signature in acute-on-chronic liver failure includes increased expression of granulocyte genes ELANE, MPO and CD177. *Scientific Reports* **11**, 18849 (2021).

47. Luo, Q. *et al.* Serum PGLYRP‑1 is a highly discriminatory biomarker for the diagnosis of rheumatoid arthritis. *Molecular Medicine Reports* **19**, 589–594 (2019).

48. Meijer, B., Gearry, R. B. & Day, A. S. The role of S100A12 as a systemic marker of inflammation. *International Journal of Inflammation* **2012**, e907078 (2012).

49. Liu, W. & Rodgers, G. P. Olfactomedin 4 expression and functions in innate immunity, inflammation, and cancer. *Cancer Metastasis Rev* **35**, 201–212 (2016).

50. Snelgrove, R. J. *et al.* A critical role for LTA4H in limiting chronic pulmonary neutrophilic inflammation. *Science* **330**, 90–94 (2010).

51. Choi, Y. J. *et al.* SERPINB1-mediated checkpoint of inflammatory caspase activation. *Nature Immunology* **20**, 276–287 (2019).

52. Jo, M. *et al.* Astrocytic orosomucoid-2 modulates microglial activation and neuroinflammation. *Journal of Neuroscience* **37**, 2878–2894 (2017).

53. Reinholz, M., Ruzicka, T. & Schauber, J. Cathelicidin LL-37: An antimicrobial peptide with a role in inflammatory skin disease. *Annals of Dermatology* **24**, 126–135 (2012).

54. Sawyer, A. J., Garand, M., Chaussabel, D. & Feng, C. G. Transcriptomic profiling identifies neutrophil-specific upregulation of cystatin F as a marker of acute inflammation in humans. *Frontiers in Immunology* **12**, (2021).

55. Zhang, L., Zhu, T., Miao, H. & Liang, B. The calcium binding protein S100A11 and its roles in diseases. *Frontiers in Cell and Developmental Biology* **9**, 693262 (2021).

56. Bi, X. *et al.* GSTP1 inhibits LPS-induced inflammatory response through regulating autophagy in THP-1 cells. *Inflammation* **43**, 1157-1169 (2020).

57. Nagaev, I., Bokarewa, M., Tarkowski, A. & Smith, U. Human resistin is a systemic immune-derived proinflammatory cytokine targeting both leukocytes and adipocytes. *PLOS ONE* **1**, e31 (2006).

58. Liu, F.-T., Yang, R.-Y. & Hsu, D. K. Galectins in acute and chronic inflammation. *Annals of the New York Academy of Sciences* **1253**, 80–91 (2012).

59. Steck, A. J., Kinter, J. & Renaud, S. Differential gene expression in nerve biopsies of inflammatory neuropathies. *Journal of the Peripheral Nervous System* **16**, 30–33 (2011).

60. Wyatt, E. *et al.* Regulation and cytoprotective role of hexokinase III. *PLOS ONE* **5**, e13823 (2010).

61. Sun, J. *et al.* Adrenomedullin 2 attenuates LPS-induced inflammation in microglia cells by receptor-mediated cAMP-PKA pathway. *Neuropeptides* **85**, 102109 (2021).

62. Han, P.-F. *et al.* Annexin A1 involved in the regulation of inflammation and cell signaling pathways. *Chinese Journal of Traumatology* **23**, 96–101 (2020).

63. Jang, B.-C. *et al.* Catalase induced expression of inflammatory mediators via activation of NF-κB, PI3K/AKT, p70S6K, and JNKs in BV2 microglia. *Cellular Signalling* **17**, 625–633 (2005).

64. Wang, Y. *et al.* Soluble resistance-related calcium-binding protein participates in multiple diseases via protein-protein interactions. *Biochimie* **189**, 76–86 (2021).

65. Cao, M.-Q. *et al.* Cross talk between oxidative stress and hypoxia via thioredoxin and HIF-2α drives metastasis of hepatocellular carcinoma. *The FASEB Journal* **34**, 5892–5905 (2020).

66. Azarova, I. E., Klyosova, E. Yu., Kolomoets, I. I. & Polonikov, A. V. Polymorphic variants of the neutrophil cytosolic factor 2 gene: Associations with susceptibility to type 2 diabetes mellitus and cardiovascular autonomic neuropathy. *Russian Journal of Genetics* **58**, 593–602 (2022).

67. Ma, J. *et al.* Glycogen metabolism regulates macrophage-mediated acute inflammatory responses. *Nat Commun* **11**, 1769 (2020).

**S13: *Lipopolysaccharide (LPS) Enzyme Immunoassay (LPS EIA) Parallelism***

**S13A**: **LPS** **experimental design.** Of 77 total serum samples used in LPS serum EIA, longitudinal samples were collected exclusively from *ex situ* animals; while single samples were obtained from 4 *ex situ* and 19 *in situ* animals.

| Number of longitudinal samples per animal | Number of animals |
| --- | --- |
| 3 | 8 |
| 2 | 15 |
| 1 | 23 |

Using a parallelism, we determined that lipopolysaccharide (LPS) was detectable in rhinoceros serum (MeanOD: 0.135, Std.Dev.0.010, CV%7.178). Four samples representing four different individual animals with diverse clinical histories were included together representing 60 uL serum per animal to make a 240 uL pooled sample. The pooled sample represented a neat pool, and serial dilutions were made as follows: 1:2, 1:4, 1:8, 1:16, 1:32, 1:64, 1:128. and standard curve obtained a LPS standard curve was recovered.

**S13B: Parallelism plate.**

**
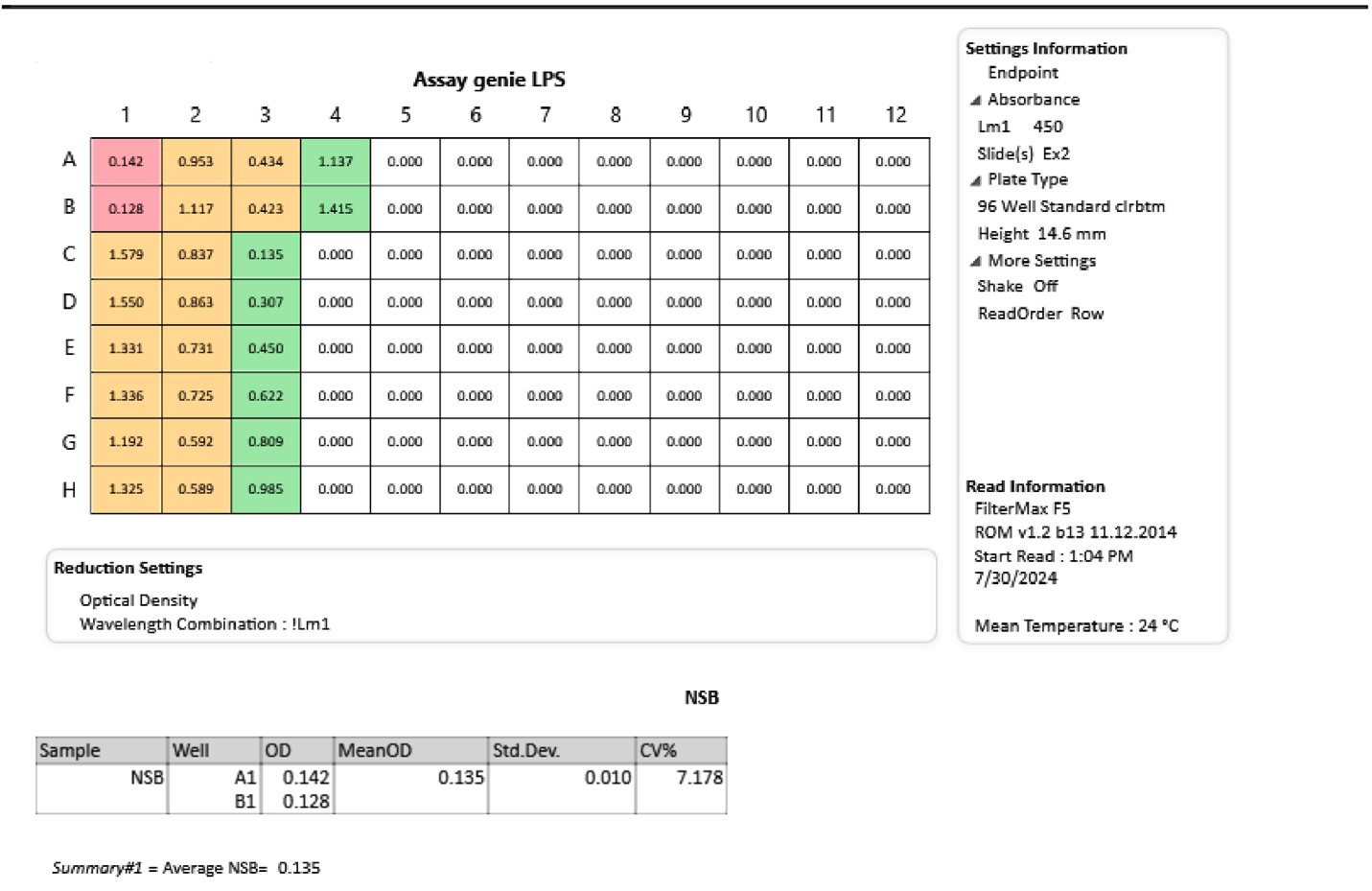
**


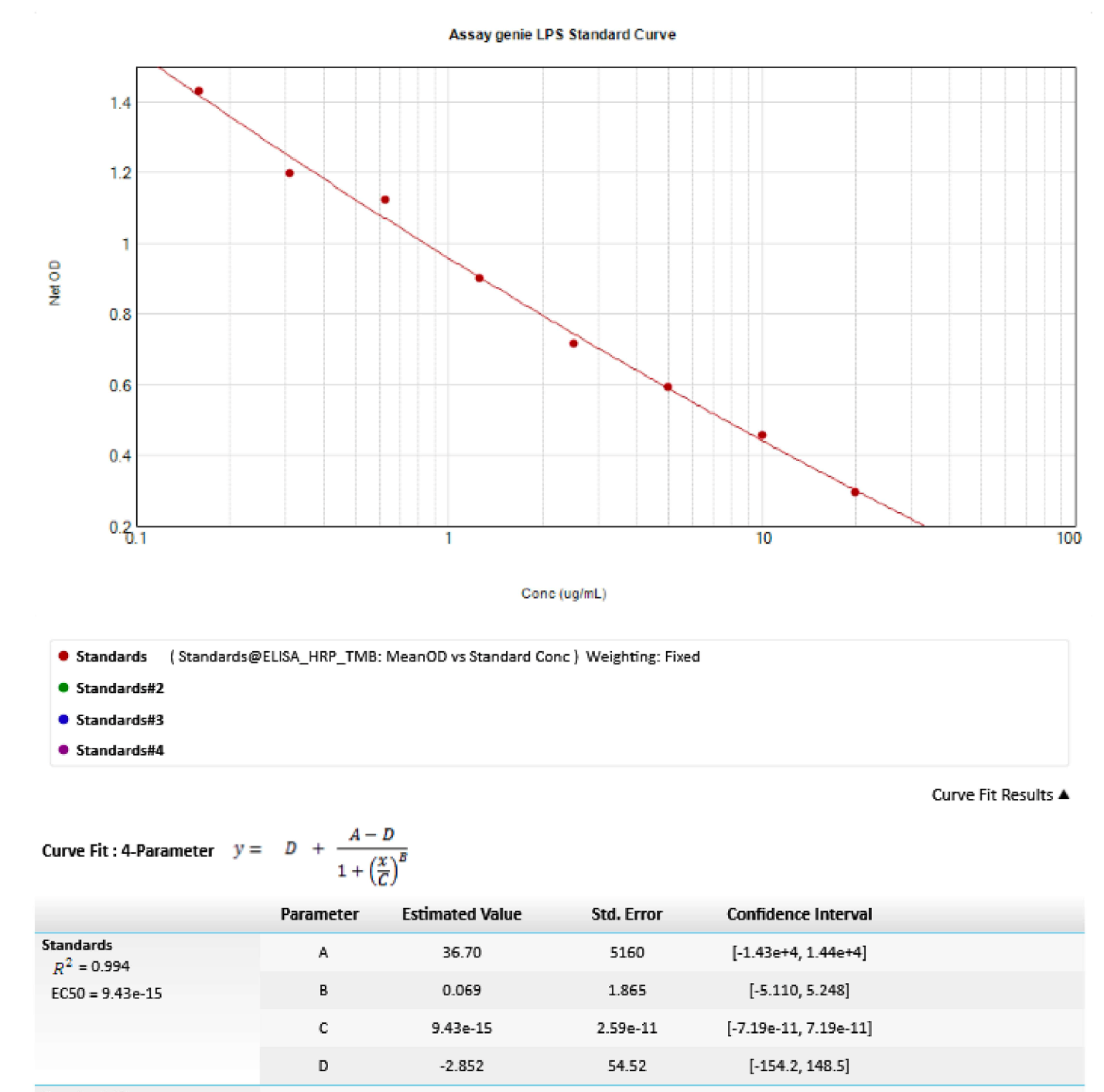


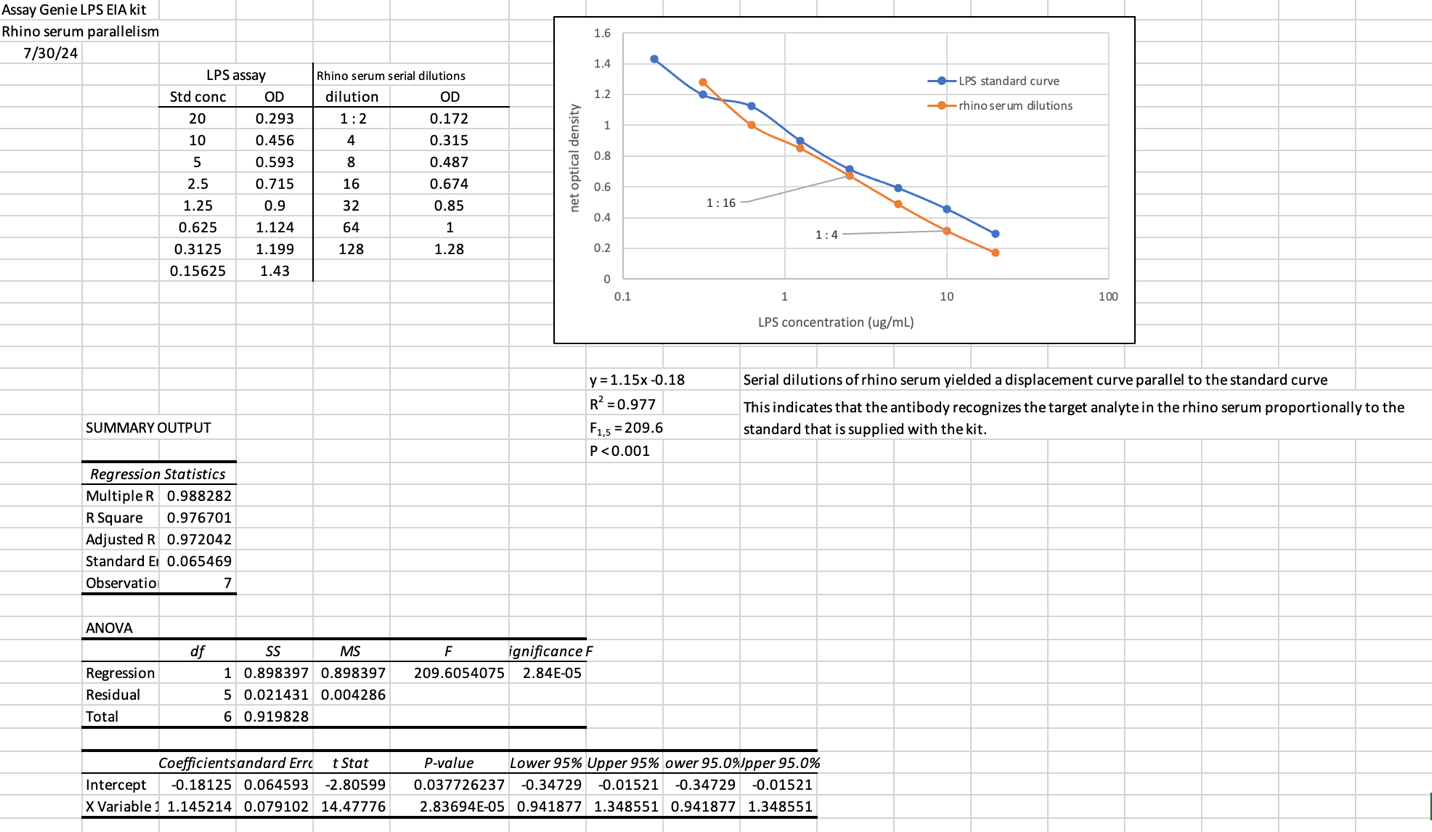


**S14: Supplementary Methods**

***Population Demographics.*** To test our hypotheses, we conducted a prospective longitudinal study on the American *ex-situ* black rhino population. We enrolled 27 black rhinos (*Diceros bicornis*) from 14 American zoological institutions in this study. We collected 80 blood samples and matching health records longitudinally. Health information was collected via an online Qualtrics survey. This population included 13 (6.7; male.female) southern black rhino (*D. b. minor*) and 14 (7.7; male.female) eastern black rhino (*D. b. michaeli*). Animals were further assigned to two groups based on health phenotypes. Animals with no history of disease were deemed “healthy”. Animals with histories of disease (chronic inflammation, ulcerative dermatitis, dental/periodontal disease, lameness, and/or metabolic disease) were classified as “inflammatory” (S2). Further, animals included in the “inflammatory” group were also reported to have clinical histories of immune dysfunction, suspected iron overload disease, anemia, renal disease, hepatic disease, chronic intermittent diarrhea, chronic intermittent nose bleeds, idiopathic hemorrhagic vasculopathy syndrome (IHVS), pododermatitis, and reproductive dysfunction (S2). Two animals could not be clearly classified into either the “healthy” or “inflammatory” phenotypes. These animals showed signs of reproductive dysfunction, but no inflammatory phenotypes. These animals were classified as “other” and omitted from the differential gene expression as a function of metadata covariates: (subspecies, sex, and health phenotype). Animals classified as “other” were included in the differential gene expression analyses as a function of consensus cluster class using an unsupervised machine learning algorithm. Wild animals were included in only the LPS experiment as PBMCs were unfortunately not available from wild animals. This population consisted of a single subspecies (*Diceros bicornis minor*) representing 9 males and 10 females. All animals resided in South Africa.

***Sample Collection.*** *Ex situ,* samples were collected opportunistically when animals were either immobilized for other medical procedures or in some cases, animals were trained via operant conditioning for voluntary participation in blood draws without use of chemical restraint. Although we did not obtain records regarding the collection site and specific protocol used for *ex situ* animals enrolled in this study, details on commonly used protocols can be found in the guidelines for rhinoceros restraint and handling^107^, authored by Dr. Michele Miller and Dr. Peter Buss. Blood products including whole blood (BD vacutainer EDTA tubes) and serum (BD vacutainer venous blood collection SST Serum Separation Tubes) were collected at participating zoological institutions and shipped cold (4^ο^C) overnight to the Smithsonian’s National Zoo & Conservation Biology Institute in Front Royal, Virginia, USA and processed the following day, approximately 24 hours post collection. Serum was aliquoted into 0.5-1.0 mL aliquots and frozen and stored at -80^ο^C until the secondary LPS ELISA experiment commenced. Whole blood was processed via the PBMC isolation protocol as outlined in the manuscript methods and supplementary sample preparation methods.

*In situ,* data collected for individual rhinoceros included date of sample collection, capture location, demographic characteristics (sex, species, and age class), and general health status of animals prior to immobilization. Health status at the time of sampling was assessed by veterinary staff as normal or abnormal. Examples of conditions associated with abnormal health status included poor body condition, visible injuries, or any treatment undergone for illness or injury at the time of sampling. Rhinos immobilized for routine dehorning procedures were considered healthy, unless one of the conditions above was recorded. Since the individual rhinos were free-ranging, prior health history was unavailable. Samples were collected from free-ranging rhinos in Kruger National Park; therefore, they were all chemically immobilized prior to sampling. Immobilization was performed by the veterinary staff according to the South Africa National Parks Animal Use and Care Committee approved Standard Operating Procedure for the Capture, Transport, and Maintenance in Holding Facilities of Wildlife. Etorphine (9.8 mg/mL, M99, Elanco, Gauteng, South Africa) and azaperone (40 mg/mL, Janssen Pharmaceutical Ltd., Halfway House, South Africa) were administered using a 3.0-mL plastic dart with 60-mm uncollared needle propelled from a compressed air rifle (DANINJECT, Skukuza, International S.A., South Africa) from a helicopter. Once immobilized, whole blood was collected from the auricular or radial vein of the rhinoceros in one EDTA (4mL) and one serum vacutainer (9mL) tube (BD Biosciences) for this study. Naltrexone (40 mg/mL, Kyron laboratories) was administered IV at 20 times the etorphine dose (in milligrams) for reversal.

*Serum amyloid A (SAA) and health status:* After determining that PBMC proteomes were not different based on metadata covariates, we attempted to validate clinical health status designations by measuring circulating SAA values among animals based on clinical metadata (“presumed healthy” vs inflammatory phenotype). We measured SAA in single time point plasma samples collected simultaneously along with other blood compartments from 25 of the 27 animals enrolled in this study. Samples were transported frozen (-20C) to the University of Miami’s Miller School of Medicine Avian and Wildlife Laboratory where concentrations of SAA were measured using a multispecies sandwich ELISA (Tridelta Diagnostics), previously validated for African rhinoceros^12-14,37^*)*.

**Sample preparation.**

**PBMC Isolation*.*** Whole blood was collected via venipuncture into EDTA blood tubes. Isolation of PBMCs was accomplished using Ficoll^TM^ density gradient (Cytiva, Marlborough, MA). All whole blood samples collected yielded PBMC pellets using a Ficoll^TM^ density gradient method, even after shipping 24-36 hours post-collection. Whole blood was diluted 1:1 (v/v) with sterile saline and mixed gently. Ficoll^TM^ (3 ml) was added to 15 ml centrifuge tubes and an equal volume of diluted whole blood was carefully layered over the Ficoll^TM^. Tubes were centrifuged (1,500 rpm; 30 min), and the PBMC layer was transferred to a fresh tube and washed twice with sterile saline. Washing was accomplished via dilution of the PBMC suspension with 3-5 ml of sterile saline, and then centrifuging (5,000 *g*; 10 min) to pellet the cells. The supernatant was discarded, and the cell pellet resuspended in fresh sterile saline and subjected to centrifugation (4,000 *g*; 5 min). The supernatant was removed. The cell pellet was covered in a layer of saline. Resulting cell pellet was stored frozen (-80°C) until mass spectrometric analysis at the Center for Applied Proteomics and Molecular Medicine at the Institute for Advanced Biomedical Research at George Mason University.

**Preparation for Mass Spectrometry.** Cell pellets (PBMCs) were thawed on refrigerated block holds (4°C) and lysed using 4 M urea. 50 µL of 4 M urea was added to each sample. The PBMC pellets were pipetted with repeated pipetting (20 times per pellet) to disturb PBMC pellet and denature proteins. Cell suspensions were centrifuged for 5 minutes at 16,000 rpm. After centrifugation, ~40 µL supernatant was transferred to a new tube. The supernatant was resuspended before protein concentration was measured using Pierce Coomassie Protein Quantification using the Eppendorf BioSpectrometer Basic (Eppendorf AG, Germany) as per manufacturer’s instructions. Protein concentrations were then normalized to ensure the same amount of protein per sample (20 µg protein/40 µL sample in 4M urea solution) before the digestion. Samples were reduced with 1M dithiothreitol (DTT) to open disulfide bonds. This was accomplished by adding 1 µL 1M DDT in the bottom of a new tube for each sample. Then a specific volume of 4M urea (calculated based on protein concentration) was added and mixed with each sample to reach 40 µL per sample. Samples containing 4M urea and DTT were transferred to a heating block (50°C) and incubated for 5 minutes. After incubation, we performed an alkylation step to digest disulfide bonds with 6 µL Iodoacetamide solution in ammonium bicarbonate (057K53011 Sigma-Aldrich, Burlington, MA) added to each sample. This step generated a stable free style group from a cystine after alkylation that could not be oxidized with other cystines to form disulfide bonds. The software used downstream modified cysteine mass in database for static modification. Next, the 4M urea was diluted to 2M urea to prevent cystine from denaturing trypsin (added in next step). For trypsin digestion, 34 µL master mix (33 µL 500 mM ammonium bicarbonate and 1 µL sequencing grade modified trypsin (0.5 ug/uL) (REF: V5117, Promega Corporation, Madison, WI) was added to each sample. Samples were incubated in a 37C water bath for 4 hours. Extracted proteins were purified using ZipTip^TM^ Pipet Tips, (CAT: ZTC18S096, Millipore-Sigma^TM^, Burlington, MA). Using ZipTip^TM^ pipet tips, we washed samples in the following order: 1) 20 µL buffer B (80% acetyl nitrile) three times, 2) 20 µL buffer A (0.1% Formic Acid) three times, 3) confirmed entire sample volume made it through tips and ejected left over solution, 4) 20 µL buffer A three times, 5) 20 µl buffer B two times. At this point, the peptide suspension was eluted into new sterile tubes. Samples were placed in a Savant^TM^ Universal SpeedVac^TM^ system UVS800DDA (Thermo Fisher Scientific, Waltham, MA) until dry (20-25 minutes). After drying, samples were stored in -20°C until the Orbitrap Exploris^TM^ mass spectrometer (CAT: BRE725539, Thermo Fisher Scientific, Waltham, MA) run.
